# The Energy Landscape Analysis of Cancer Mutations in Protein Kinases

**DOI:** 10.1371/journal.pone.0026071

**Published:** 2011-10-06

**Authors:** Anshuman Dixit, Gennady M. Verkhivker

**Affiliations:** 1 Department of Pharmaceutical Chemistry, School of Pharmacy, The University of Kansas, Lawrence, Kansas, United States of America; 2 School of Computational Sciences and Crean School of Health and Life Sciences, Schmid College of Science and Technology, Chapman University, Orange, California, United States of America; 3 Department of Pharmacology, University of California, San Diego, La Jolla, California, United States of America; University of Akron, United States of America

## Abstract

The growing interest in quantifying the molecular basis of protein kinase activation and allosteric regulation by cancer mutations has fueled computational studies of allosteric signaling in protein kinases. In the present study, we combined computer simulations and the energy landscape analysis of protein kinases to characterize the interplay between oncogenic mutations and locally frustrated sites as important catalysts of allostetric kinase activation. While structurally rigid kinase core constitutes a minimally frustrated hub of the catalytic domain, locally frustrated residue clusters, whose interaction networks are not energetically optimized, are prone to dynamic modulation and could enable allosteric conformational transitions. The results of this study have shown that the energy landscape effect of oncogenic mutations may be allosteric eliciting global changes in the spatial distribution of highly frustrated residues. We have found that mutation-induced allosteric signaling may involve a dynamic coupling between structurally rigid (minimally frustrated) and plastic (locally frustrated) clusters of residues. The presented study has demonstrated that activation cancer mutations may affect the thermodynamic equilibrium between kinase states by allosterically altering the distribution of locally frustrated sites and increasing the local frustration in the inactive form, while eliminating locally frustrated sites and restoring structural rigidity of the active form. The energy landsape analysis of protein kinases and the proposed role of locally frustrated sites in activation mechanisms may have useful implications for bioinformatics-based screening and detection of functional sites critical for allosteric regulation in complex biomolecular systems.

## Introduction

Rapid and efficient communication of long-range conformational changes in proteins plays a vital role in allosteric regulation of biological systems[**1]**, [2****]. Recent seminal reviews of protein allostery have emphasized a central role of cooperativity and the notion that catalysis and allostery may emerge via common communication routes [**3]**, [4****]. Modeling of allosteric transitions in biological molecules has been significantly advanced by the development of elastic network models and normal mode analysis approaches [**5]**-[22****]. Elastic network models of protein dynamics and signal propagation theory have allowed for a quantitative analysis of long-range allosteric protein interactions [**13]**-[16****]. Sequence-based evolutionary analysis [**23]**, [24****] and structure-based approaches [**19]**, [20], [25]-[27****] have demonstrated that allosteric pathways in proteins may be formed through interactions of evolutionary conserved and sparsely connected clusters of residues that are energetically coupled to mediate long-range communication. A comprehensive analysis of allosteric mechanisms has led to a unified view of allosteric regulation that implies the existence of preexisting conformational states and multiple communication pathways on the conformational landscape [**28]**-[32****]. Energy landscape theories and simplified energy models have provided a robust theoretical framework to elucidate fundamental aspects of protein structure, dynamics and allosteric regulation [**33]**-[43****]. According to the modern energy landscape theory, random sequences have rugged landscapes with many local minima due to severe conflicting interactions (a phenomenon termed “frustration”) and, as a result, the prevalence of structurally alternative yet energetically similar conformations. The energy landscape models have also suggested that protein-like sequences may have evolved to partially eliminate frustrated interactions between amino acids and have smoothed (“funnel-like”) landscapes to ensure fast folding to their thermodynamically stable native structures. This has become known as the ‘principle of minimal frustration’ [**44]**, [45****]. The funneled-like nature of the energy landscapes for natural proteins implies that the conformations that are structurally similar to the native state are also low in energy, and the native state interactions are minimally frustrated [**33]**-[45****]. A generalized view of allosteric regulation based on the energy landscape theory (often termed as a “conformational selection model”) suggests that a protein may function in a dynamic equilibrium of structurally different conformational states, whereby the effect of binding or mutation can be propagated over long distances by cooperatively shifting the equilibrium towards a functionally relevant conformation [**46]**-[52****]. The "old" view (induced fit mechanism) and the "new" view (conformational selection mechanism) of protein allostery appeared not to be mutually exclusive but rather complementary in rationalizing allosteric mechanisms at the molecular level [**53]**-[56****]. Physics-based simulation approaches have provided a compelling evidence of coupling between collective motions and local structural changes as an important underlying principle of allosteric communication in biomolecules [**53]**-[60****]. Thermodynamics-based approaches have further linked global and local structural perturbations with free energy changes of allosteric coupling in mechanisms conformational switching [**61]**-[64****]. Moreover, the energy landscape models have suggested that long-range cooperativity of protein-protein interactions during allosteric transitions may favor a combination of the population-shift and induced-fit mechanisms, whereas short-range, allosteric binding of proteins with inhibitors could often proceed via the population-shift mechanism [**65]**-[72****].

Ferreiro and Wolynes [**69**] have recently advanced the energy landscape theory by combining biophysical modeling and structural bioinformatics analyses of local protein interactions that are fundamental for folding, binding and allosteric regulation. According to this model, minimally frustrated landscapes of protein networks may have evolved to acquire the ability for regulation via cooperative allosteric changes. The proposed method has quantified the degree of spatial local frustration in proteins using a local version of the global gap criterion formulation of the minimal frustration principle [**33]**-[45****]. This model introduced a local frustration metric termed “configurational frustration index” as a measure of local stabilization for an individual native pair with respect to a set of structural decoys generated by perturbing both the identities and location of the interacting amino acids [**69]**, [70****]. According to this criterion, if the interaction energy of a native pair of residues is sufficiently stabilizing as compared to the set of structural decoys, this residue pair is designated as “minimally frustrated’’, otherwise the interactions may be classified as either “neutral” or “locally frustrated”. It is worth noting that the principle of minimal frustration does not require a complete elimination of locally stable alternative structures. A certain degree of local frustration is always present in an otherwise largely unfrustrated protein structure and may have arisen from evolutionary requirements to adapt protein dynamics for specific functions [**43**].

The analysis of locally frustrated protein regions using a non-redundant set of 314 monomeric protein domains and a curated set of nonredundant dimeric complexes has shown that the locally frustrated sites correspond to the regions involved in binding with other macromolecules and ligands and could often collocate with the functional groups prone to large structural changes [**69]**, [70****]. Wolynes and coworkers have recently surveyed a curated database of allosteric proteins with known inactive and active crystal structures and have demonstrated that allosteric protein domains are connected by a web of minimally frustrated interactions, while highly frustrated residues could be preferentially clustered near the protein surface [**71]**, [72****]. According to this study, minimally frustrated regions in allosteric proteins domains constitute nearly 40% of the total contacts, with about 10% of the total interactions considered to be “highly frustrated”, and the remainder of interactions attributed to the “'neutral” category.

Protein kinases are signaling switches with a conserved catalytic domain that phosphorylate protein substrates and play a critical role in cell signaling pathways [**73]**-[82****]. Protein kinase genes constitute ∼2% of all genes in human genome and this protein family consists of more than 500 diverse members. The crystal structures of human protein kinases include 167 unique human protein kinase domains and 170 kinases, considering closely related orthologues (http://www.sgc.ox.ac.uk/research/kinases/). Structural studies of protein kinase catalytic domain structures and regulatory protein complexes have revealed distinct scenarios by which kinases can control a dynamic equilibrium between structurally similar active and highly specific inactive kinase states - a structural hallmark of the kinase domain critical for its normal function [**83]**-[89****]. Allosteric regulation may be achieved via different mechanisms including inhibitor-induced stabilization of the specific inactive conformation in ABL [**90]**-[95****], BRAF [**96**], KIT [**97**], PDGFR, P38 [**98**], PI3K kinases [**99**] and binding to the allosteric myristoyl-binding pocket in ABL [**100]**-[103****]. Protein kinase activation can be also regulated via formation of structurally diverse regulatory complexes most notably exemplified for ABL [**104]**, [105****] and EGFR kinases [**106]**-[109****], yet a unifying structural mechanism associated with asymmetric tyrosine kinase arrangements in regulatory complexes could underlie the activation mechanism of the entire EGF protein family [**110]**-[113****]. A steady progress in understanding of protein kinase mechanisms has fueled a considerable effort to discover and design selective ATP-competitive and allosteric inhibitors targeting specific forms of cancer, kinase cancer mutants and associated targeted pathways [**114]**-[116****].

Abnormal activation of regulation in protein kinases is a dominant source of tumor-associated somatic mutations. Structural and mutagenesis investigations of ABL [**93]**-[95****] and EGFR kinases [**117]**-[119****] have revealed structural divergence of the kinases in response to activating mutations. Kinome-wide bioinformatics studies have contributed to the identification of conserved sequence motifs harboring disease-associated and cancer mutations, suggesting that a significant number of oncogenic cancer mutations could form structurally conserved mutational hotspots within the kinase catalytic core [**120]**-[123****]. Computer simulation studies have investigated molecular mechanisms of protein kinase activation in c-Src [**124]**-[127****], adenylate kinase [**128**], ABL [**129**], CDK5 [**130**], KIT [**131**], RET, MET [**132**] and EGFR kinase [**133]**-[135****]. Multi-scale simulation studies of conformational transitions in the normal and oncogenic forms of ABL and EGFR kinases have indicated that the impact of the oncogenic mutants may spread beyond the immediate site of mutation leading to global allosteric changes [**134**]. Most recently, computational modeling of allosteric regulation has revealed organizing principles of mutation-induced activation in ABL and EGFR kinases, which may be determined by a dynamic coupling between structurally rigid αF-helix and conformationally adaptive αI-helix and αC-helices [**136**]. These structural elements form a dynamic network of efficiently interacting functional regions that may universally control the long-range interdomain communication and allosteric activation in protein kinases. The energy landscape studies have previously suggested that localized frustration may be connected with allosteric conformational changes in proteins [**69]**-[72****].

Collectively, computational studies have suggested that molecular mechanisms of allosteric regulation in protein kinases can be described using models of mutation-induced modulation of the conformational landscape and conformational selection principles of the thermodynamically relevant states. In this work, kinome-based structural bioinformatics analysis and biophysical modeling of protein kinase structures were employed to characterize and quantify the interplay between oncogenic kinase mutations and locally frustrated sites as potential catalysts and mediators of kinase activation. The results of this study suggest that the energy landscape effect of oncogenic mutations may be allosteric in nature, eliciting global changes in the spatial distribution of highly frustrated residues. We show that cancer mutations could act by simultaneously perturbing the network of minimally frustrated interactions in the inactive kinase state, while reducing local frustration and restoring allosteric interactions in the active kinase form. Hence, locally frustrated sites in the catalytic core may serve an important functional role by enabling mutation-induced conformational transitions towards the constitutively active kinase conformation.

## Results

### The Energy Landscapes and Local Frustration in Protein Kinases

**Figure 1:**
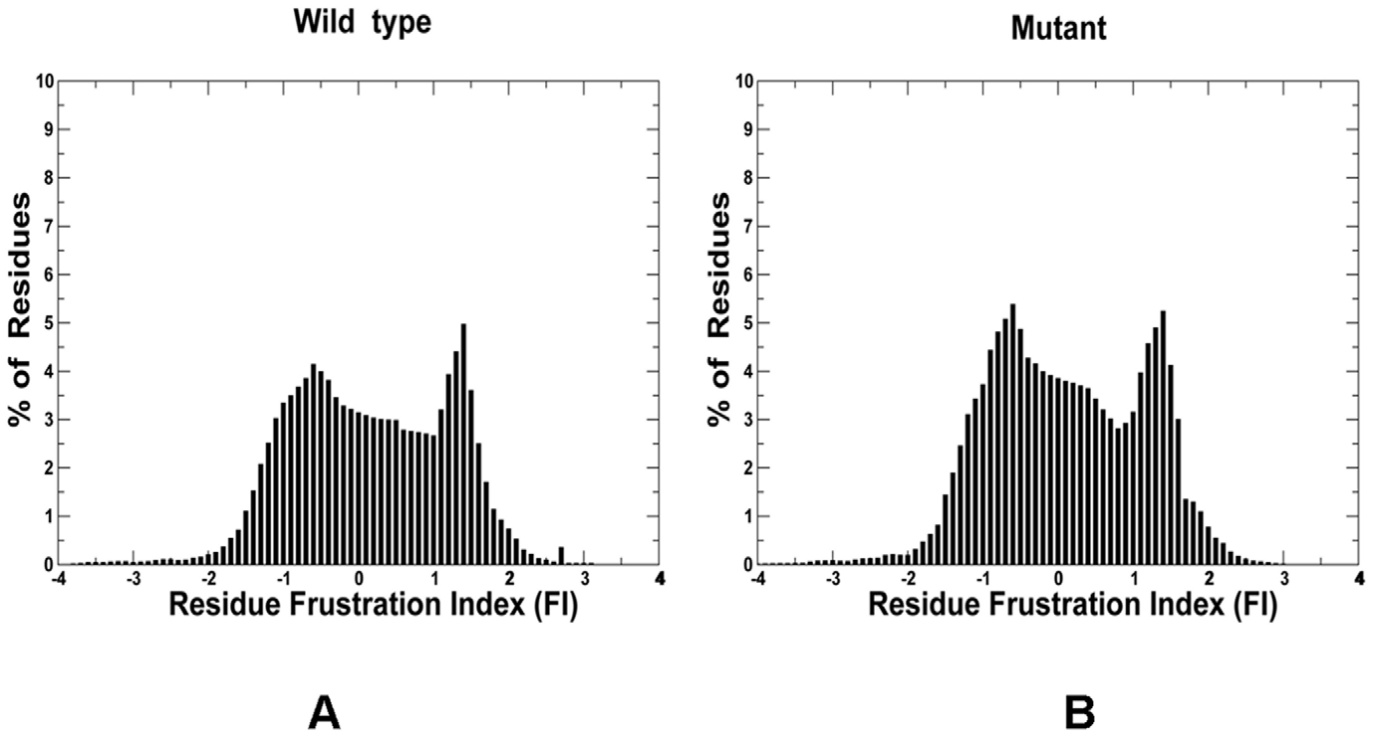
The histogram of a residue-based frustration index values in the catalytic domain from a kinome-based analysis. (A) Wild-type kinases and (B) Mutant kinases.

In the present study, we combined molecular dynamics (MD) simulations of protein kinases with the energy landscape analysis to characterize the role of local frustration as an important factor associated with allostetric kinase activation. From the energy landscape perspective, the mechanistic features of the activation transitions should be determined by the structural topology of the kinase domain fold and therefore could capture salient aspects of the activating mechanism. To investigate the role of local frustration in conformational transitions between structurally different functional states, we surveyed the local frustration profiles in protein kinase structures and characterized the network of minimally frustrated interactions responsible for structural stability of the kinase catalytic core. We also located and characterized clusters of locally frustrated sites where the minimal frustration principle could be violated. The change in the configurational frustration index upon mutation can provide a quantitative measure of a tendency to bring about a conformational change in the protein. The effect of kinase cancer mutations on local frustration profiles allowed us to quantify how mutation-induced redistribution of locally frustrated residues can promote allosteric transitions between structurally distinct functional states. The configuration frustration index could measure the relative stability of a particular native contact relative to the set of all possible contacts in that location, thus allowing to classify the individual native contacts in the protein structure according to their frustration level. A kinome-wide examination of the configurational frustration index computed for a large number of protein kinase crystal structures (**Table S1** in **File S1**) revealed that the typical values may range between −4 to +4. The overall residue-based distribution of a frustration index for the wild type (WT) kinases was biased towards minimally frustrated residues with the positive values of the frustration index (**Figure 1A**). The distribution also displayed a smaller shallow peak corresponding to locally frustrated residues with the frustration index in the range of −0.6 to −0.7 units. The impact of mutations resulted in a subtle yet noticeable change in the distribution local of frustration in the catalytic core, revealing a second equally important peak around -1.0 value. Hence, the overall distribution was almost evenly divided between the minimally frustrated and frustrated residues, leaving fewer residues at the neutral status (**Figure 1B**).

**Figure 2:**
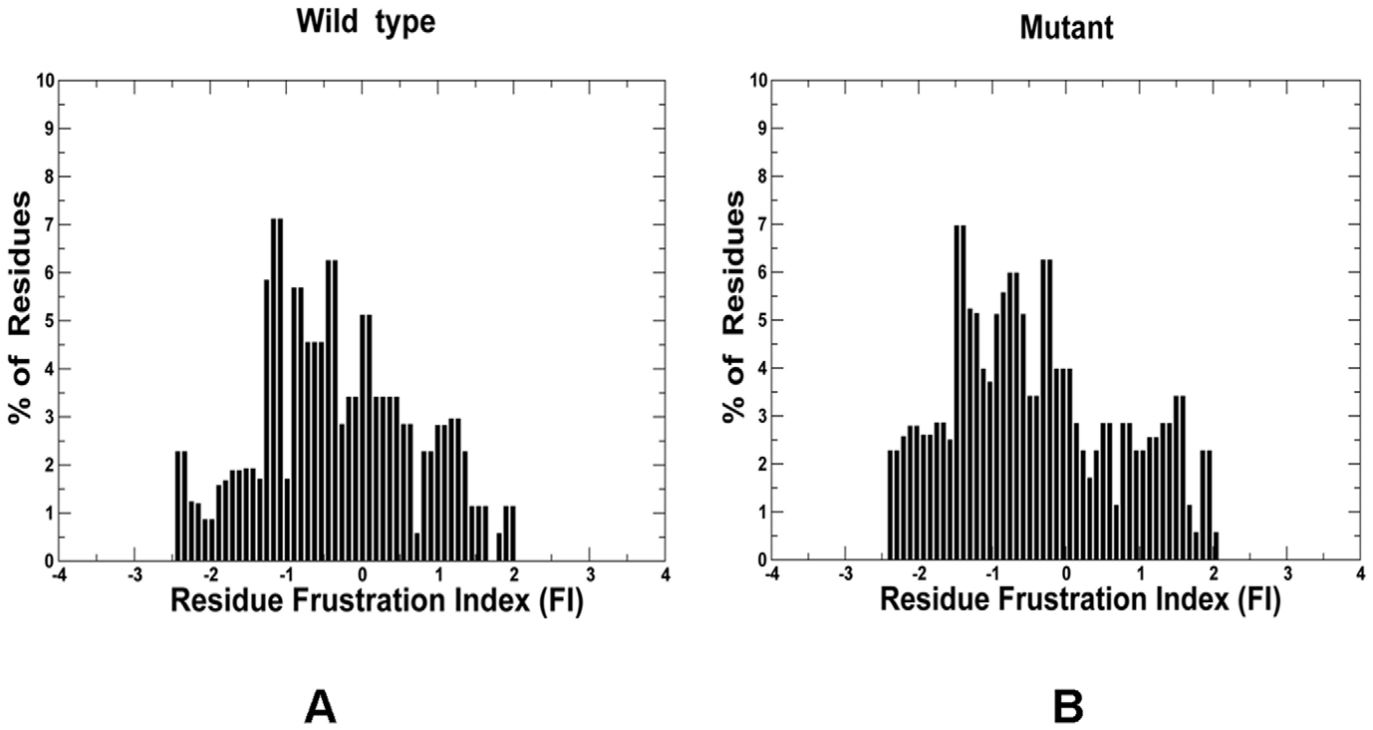
The histogram of a residue-based frustration index values in the kinase catalytic domain from a statistical analysis of dominant kinase oncogenes. (A) Wild-type kinases and (B) Mutant kinases. A set of employed kinase oncogenes included ABL, EGFR, BTK, KIT, MET, BRAF, and RET kinases. The analysis included mutants of these kinase genes with high oncogenic potential according to the frequency profiles in the mutational samples (>5) obtained from the COSMIC repository [153].

We focused then on the local frustration analysis performed for a subset of protein kinase genes (ABL, EGFR, BTK, KIT, BRAF, MET, and RET) that account for the vast majority of highly oncogenic mutations in the catalytic domain (**Table S2** in **File S1**). These protein kinase genes were chosen for a more detailed analysis because of the wealth of structural and functional information that provided complementary experimental data for validation of our models. More importantly, however, a diverse repertoire of activating and drug resistant mutations in these kinases genes represent critical cancer culprits that could frequently contribute to a state of oncogene dependency in a variety of cancers. The distribution of local frustration in these kinase genes, as measured by the configurational frustration index, revealed a distinct pattern where the peaks were noticeably shifted towards more frustrated residues for both the WT and mutant kinases (**Figure 2**). The percentage of minimally frustrated interactions in the catalytic core accounted for more than 40% of the total contacts, with about 15-20% of the interactions be considered as frustrated and the remainder neutral. This analysis generally agreed with the reported distribution of frustrated regions and partition of minimally and highly frustrated residues in proteins [**69]**, [70****]. However, the average fraction of locally frustrated residues was higher in protein kinases than the one reported for small monomeric proteins. Hence, our data suggested that conformational landscapes of kinase oncogenes may be characterized by an increased level of local frustration and protein mobility.

**Figure 3:**
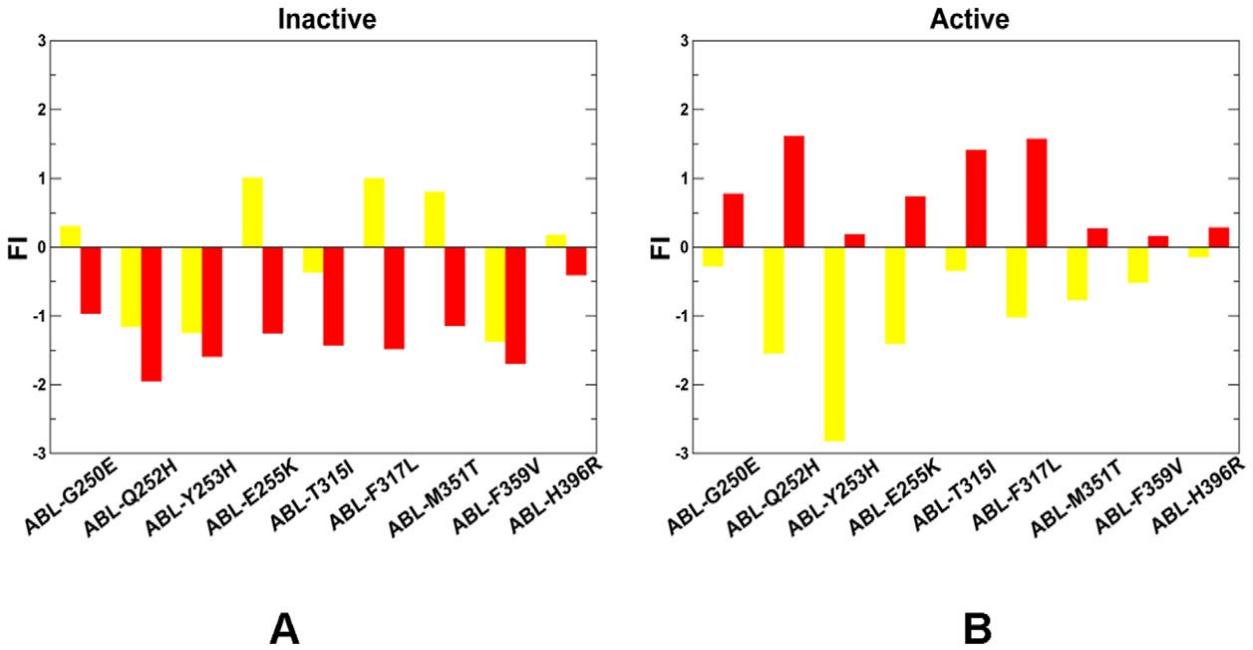
The effect of oncogenic mutations on local frustration in the ABL kinase. The residue-based frustration index values are shown for a set of oncogenic ABL kinase mutants in the inactive (A) and active forms (B). The frustration index values are shown in filled yellow bars for the wild-type kinase form and in red filled bars for the mutant forms. The analysis was performed on the unbound form of the crystal structures of ABL in the inactive form (PDB ID 1IEP) [**90**] and active form (PDB ID 1M52) [**91]**, [92****].

**Figure 4:**
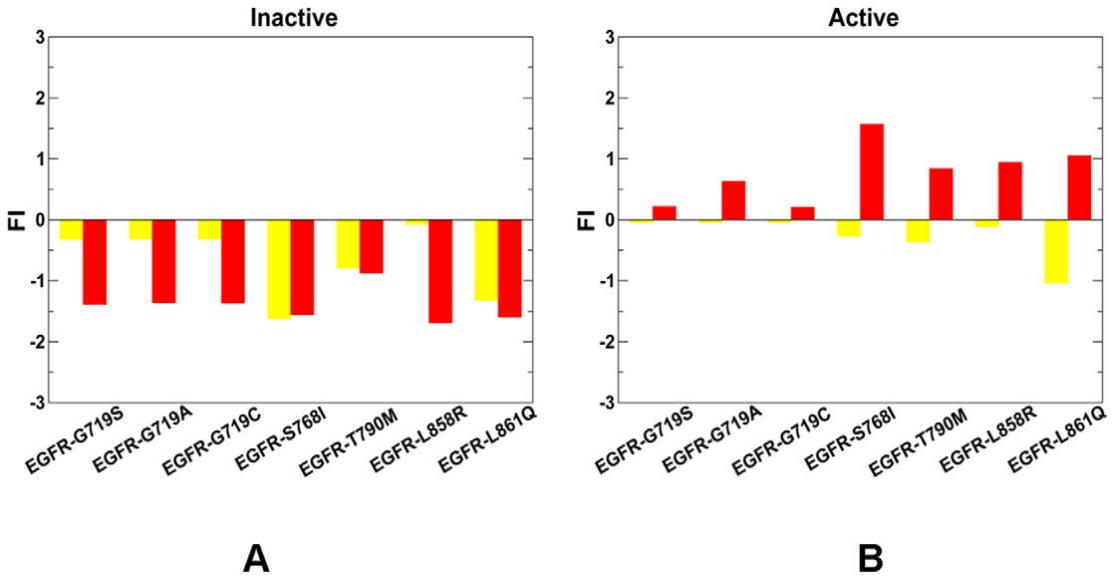
The effect of oncogenic mutations on local frustration in the EGFR kinase. The residue-based frustration index values are shown for a set of oncogenic EGFR kinase mutants in the inactive (A) and active forms (B). The frustration index values are shown in filled yellow bars for the wild-type kinase form and in red filled bars for the mutant forms. The analysis was performed on the unbound form of the crystal structures of EGFR in the inactive form (PDB ID 1XKK) [**117**] and active form (PDB ID 2J6M) [**118**].

Based on this analysis, we proposed that the spatial distribution of local frustration in protein kinases may be regulated and readily changed by oncogenic mutations. According to our conjecture, activating kinase mutations could amplify the local frustration in the inactive state, while eliminating (or partly removing) locally frustrated sites in the active state. As result, mutation-induced redistribution of local frustration in protein kinase structures may contribute to the molecular mechanisms that control kinase activity by altering the dynamic equilibrium between functional kinase forms. To verify this hypothesis, we analyzed changes in the local frustration profiles for a representative set of highly oncogenic ABL (**Figure 3**) and EGFR kinase mutants (**Figure 4**) in both inactive and active states. We observed that highly oncogenic mutations may indeed cause an increase in the local frustration of mutated residues in the inactive autoinhibitory state of ABL (PDB ID 1IEP) [**90**] and EGFR (PDB ID 1XKK) [**117**] (**Figures 3** **,** **4**). Hence, kinase mutations with a high oncogenic potential may destabilize the autoinhibited kinase form. Importantly, oncogenic mutations could partly alleviate local frustration in the active kinase state (**Figures 3** **,** **4**). A more extensive minimally frustrated network of interactions rigidifies the active form of the catalytic domain for ABL (PDB ID 1M52) [**91]**, [92****] and EGFR (PDB ID 2J6M) [**118**]. Although the crystal structures employed in our study (**Table S1** in **File S1**) were mostly solved in the unbound form, there were some structures in the studied set (particularly ABL and EGFR kinases) that were originally crystallized in complexes with ATP or small molecule inhibitors. Since ATP binding could potentially increase structural rigidity of the catalytic domain, the local frustration analysis that included structures with a removed ATP may produce artificial changes in the frustration index for binding site residues. We evaluated the overall statistical distribution of the configurationally frustration index using crystal structures with bound molecules. The effect was found to be rather negligible and the resulting distribution was virtually indistinguishable from the one shown in **Figure 1** **.** Indeed, a significant fraction of the protein kinase residues involved in binding site interactions belong to the structurally rigid hinge region which is a minimally frustrated element of the catalytic core and as such robust to minor perturbations of interactions. However, we found some interesting small variations in the local frustration profiles of ABL (**Figure S1**) and EGFR kinases (**Figure S2**), which were observed for oncogenic mutations in the glycine-rich P-loop (ABL-G250E, ABL-Q252H, ABL-E255K, EGFR-G719S, EGFR-G719A, EGFR-G719C). It is known that the P-loop in ABL kinase may be stabilized in the Imatinib-bound inactive structure, which may explain the increased local frustration upon P-loop mutations in the inactive state (**Figures S1, S2**). Interestingly, these point mutations are known to impair the binding of Imatinib (Gleevec) to ABL by shifting the thermodynamic equilibrium towards the active form incompatible with the inhibitor binding [**114]**-[116****]. Our data suggested that mutation-induced local frustration in the inhibitor-bound inactive kinase state may partly contribute to initiating a population shift between functional forms. We also analyzed the distribution and structural partition of minimally frustrated and locally frustrated regions in the ABL kinase (**Figure S3**). A dense network of minimally frustrated residues was found in the structurally rigid core of the catalytic domain (connected by green lines). This minimally frustrated web was formed by structurally conserved αF-helix and αE-helix. In contrast, the clusters of locally frustrated residues (connected by red lines) assembled on the protein periphery, including the αC-helix, activation loop, the P+1 loop in the C-terminal lobe. As the autoinhibiting interactions released in the active form, protein kinases could become more flexible with a considerable degree of residual local frustration. This was reflected in the increased presence of locally frustrated residues connected by red lines in the αC-helix, and the C-terminal lobe of the active ABL (**Figure S3B**).

### Local Frustration and Protein Flexibility

We also investigated a relationship between local frustration and protein flexibility of kinase structures. In our previous studies, we have characterized the conformational landscapes of ABL, EGFR, RET and MET kinases as well as various cancer mutants using MD simulations of the Apo kinase and complexes with ATP and small molecule inhibitors [**132]**, [134], [136****]. Here, we compared the results of local frustration analysis with the kinase flexibility profiles, which were inferred from MD simulations and evaluated using the root mean square fluctuations (RMSF) of the catalytic domain residues. In particular, MD studies of ABL and EGFR kinases in the normal and oncogenic states displayed a high local flexibility in the lower portion of the activation loop [**134]**, [136****]. Similarly, the bundle of α-helices in the C-terminal, which represented the densest cluster of minimally frustrated residues (**Figures S4,S5**), also demonstrated the smallest variation in the RMSF values - a characteristic of structurally rigid protein core [**134**]. The local frustration profiles also matched up nicely with the B-factors of the protein kinase residues. An example of such comparative analysis was detailed for the EGFR-WT in the active form (**Figure S5**). A robust correlation was found between the residue-based local frustration index and the B-factor values (**Figure S5 D**). We also observed that the highly frustrated EGFR residues corresponded to the conformationally mobile regions with the higher B-factor values. To further illustrate these findings, we performed structural mapping of the average B-factors onto a set of inactive (**Figure S5 A**) and active structures (**Figure S5 B**) of ABL, EGFR, BTK, KIT, BRAF, MET, and RET kinases. Additionally, locally frustrated residues were also mapped onto the catalytic core. The locally frustrated sites corresponded to protein regions with the increased thermal mobility and overlapped with the protein residues of higher B-factors.

The analysis of protein kinase flexibility has also demonstrated that conformational changes in functionally important kinase regions may be allosterically coupled and highly correlated. More specifically, we found evidence of highly correlated protein motions and allosteric coupling of the αC-helix and activation loop with all other kinase regions (**Table S3** in **File S1**). Interestingly, the αC-helix and the activation loop represented two most highly coupled protein kinase regions. Other highly correlated segments of the catalytic domain included (a) the hinge region and catalytic loop, and (b) the P-loop and activation loop. These findings consistent with our recent analysis of collective motions in ABL and EGFR regulatory complexes that manifested in “breathing” rigid body movements of the catalytic core coupled with the fluctuations of the P-loop, activation loop, αC-helix and the αG-helix of the C-terminal [**136**]. Numerous structural biology studies have also indicated a central involvement of the αC-helix and activation loop in allosteric coupling that control regulation of protein kinase activity [**110]**-[113****].

### Allosteric Effect of Oncogenic Mutations on Local Frustration

**Figure 5:**
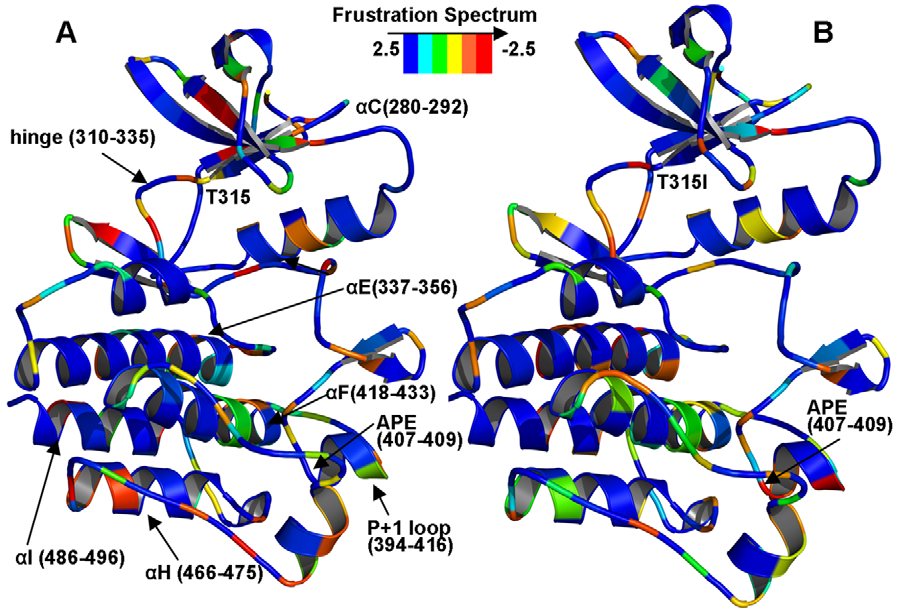
The effect of oncogenic mutation on spatial distribution of local frustration in the ABL kinase. The spatial distribution of local frustration in the inactive forms of ABL-WT (A) and ABL-T315I (B). The color sliding scheme of local frustration ranges from minimally frustrated (shown in blue) to highly frustrated (shown red). The key functional regions of the protein kinase along with the respective range of protein residues are referred to by arrows. Structural mapping of local frustration on the ABL kinase catalytic core is shown for the inactive ABL-WT structure (PDB ID 1IEP) [**90**]. The effect of T315I on the inactive ABL structure was evaluated via structural modeling detailed in the Materials and Methods section. The Pymol program was used for visualization of protein kinase structures and the local frustration mapping (The PyMOL Molecular Graphics System, Version 1.2r3pre, Schrödinger, and LLC).

We investigated if spatial distribution of local frustration may present initiation points for global conformational changes and whether the effect of oncogenic mutations on the local frustration would be local or allosteric. If the effect of oncogenic mutations was local, it would cause only local perturbations and result in the negative values of the frustration index for residues in the immediate proximity of the mutational site. However, if the effect of oncogenic mutations was global, the spatial distribution of highly frustrated residues may be allosterically affected and result in noticeable changes at the remote from the mutational site regions. A comparison of locally frustrated residues mapping in the ABL-WT and ABL-T315I mutant revealed subtle yet relevant changes, where most of the effected residues were remote from the mutational site (**Figure 5**). We observed that the gate-keeper mutation in the inactive kinase form may allosterically perturb structural rigidity of the catalytic core and increase local frustration of the αF-helix, αE-helix, and αC-helix regions. Our findings corroborated with a hydrogen exchange mass spectrometry (HX MS) study of ABL kinase [**100**], indicating that the effect of the ABL-T315I mutation could result not only in local conformational disturbances near the αC-helix, but also allosterically change protein flexibility in the distant from mutation protein regions. The changes in the local frustration induced by ABL-T315I mutation in the inactive kinase could be illustrated in examples presented in **Figures S6, S7**. While frustration plots of ABL-WT and ABL-T315I were generally similar, there were some changes in the red line clusters connecting Asp-381 of the DFG motif to Glu-286 which makes an important hydrogen bond with Lys-271 (**Figure S6**). Another change could be noted in the anti-parallel β-sheet from the lower part of the activation loop (**Figure S7**). In this region a few of the residues become highly frustrated upon mutation as evident by red lines connecting residues Tyr-393, Ala-395 and Pro-402.

Importantly, partial unfolding of the anti-parallel β-sheet at the lower end of the activation loop was previously determined as a prerequisite for stabilization of the intermediate Src-like structure and a common mechanistic feature of the ABL and EGFR activation pathways [**134**]. In the Src-like conformation, αC-helix was rotated and moved out of the active site (αC-helix-Glu-out position), the DFG motif flipped in the intermediate DFG-in position, the anti-parallel β-sheet from the lower part of the activation loop unfolded and the P-loop moved in towards the active site. These structural changes were accompanied by a concerted breakage of the K271-E286 ion pair and formation of the E286-R386 salt bridge. A conserved salt bridge between the K271 and E286 is a structural hallmark of the inactive and active forms of ABL, while it is absent in the Src-like inactive structure. The formation and breakage of this critical interaction coupled with the conformational changes in the DFG motif are critical structural features underlying mechanisms of kinase activation [**134**]. A mutation-induced development of local frustration in the DFG motif and the β-sheet of the activation loop could present the “initiation cracking points” [**65]**-[72****] that would likely to perturb the inactive kinase form and facilitate conformational transitions between alternative kinase states. These results agree with the energy landscape analysis of adenylate kinase [**66**], in which the high stress region in the activation loop may “crack” or locally unfold releasing the strain and thus catalyzing a global conformational transformation. According to the “cracking” model [**137]**-[139****], allosteric conformational changes can be triggered by the increased local frustration causing thermodynamic destabilization of a protein region via local unfolding. We found that mutation-induced perturbation of minimally frustrated interactions and amplification of protein flexibility in the inactive kinase state could be compounded by a partial reduction of local frustration and structural consolidation of the active kinase. Collectively these effects may present a feasible mechanism of kinase activation by cancer mutations via exploiting redistribution of local frustration to facilitate conformational transitions and enhance the thermodynamic stability of the constitutively active kinase. The proposed model is consistent with the energy landscape ideas according to which low local stability should accompany high local frustration and locally frustrated regions may act as local cracking points or specific hinges during allosteric changes [**65]**-[72****,**137–139**].

### Oncogenic Mutations as Allosteric Switches of Local Frustration

**Figure 6:**
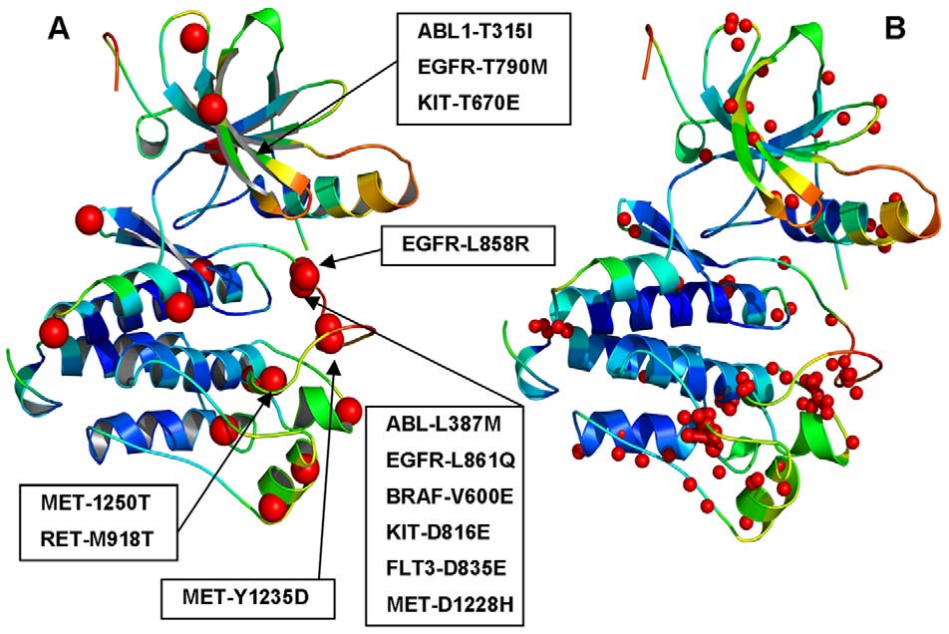
The spatial distribution of highly oncogenic mutations and highly frustrated residues in the kinase catalytic core. The kinase mutations with known high oncogenic potential were mapped onto kinase catalytic domain (**A**). Structurally conserved hotspots of kinase cancer mutations are annotated by large red spheres and their location is indicated by arrows in (**A**). The locally frustrated sites (FI<-2.0) mapped onto kinase catalytic domain and depicted as small red spheres (B). The crystal structure of EGFR-WT in the active form (PDB ID 2J6M) [**118**] was used as a template for structural mapping. The Pymol program was used for visualization of protein kinase structures and the local frustration mapping (The PyMOL Molecular Graphics System, Version 1.2r3pre, Schrödinger, and LLC.).

**Figure 7:**
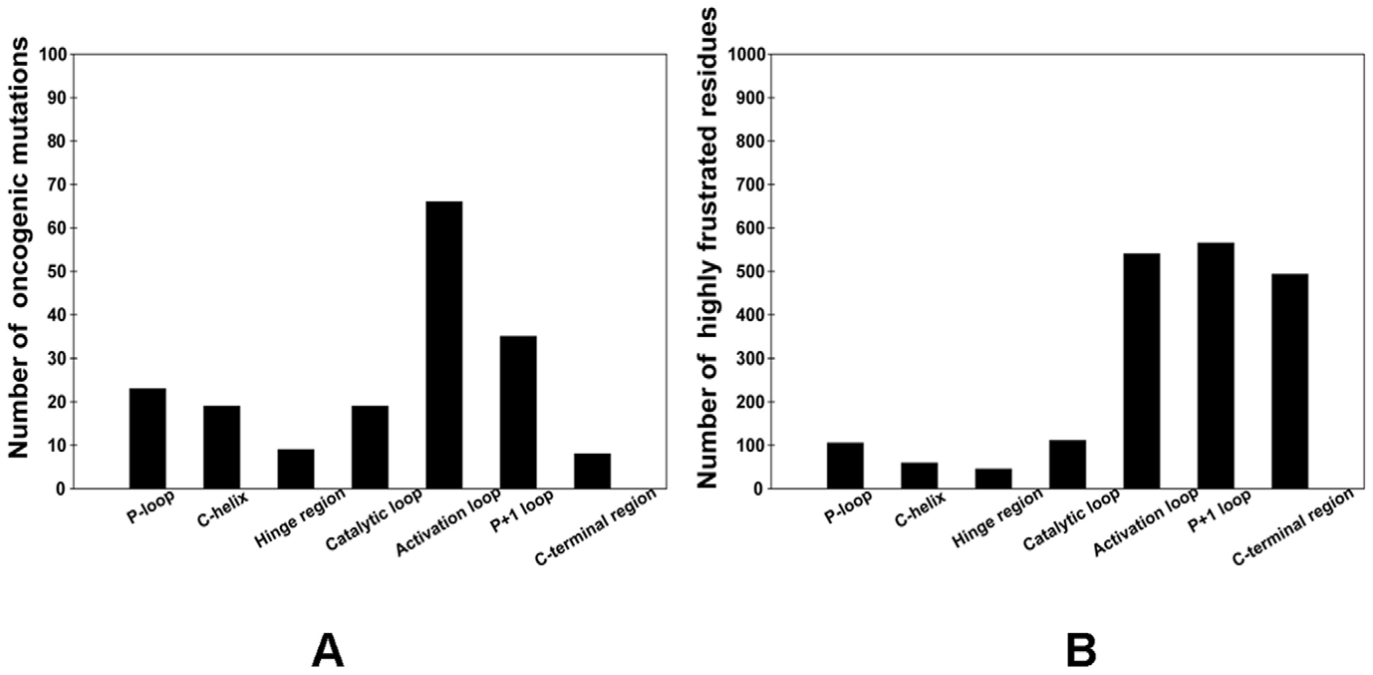
The distribution of oncogenic mutations and locally frustrated sites at each structural region in the catalytic core. The distribution of cancer mutations (A) and the distribution of local frustration (B) across catalytic core subdomains. The analysis is performed based on known oncogenic mutations in the ABL, EGFR, BTK, KIT, BRAF, MET, and RET kinase genes. The residue ranges of the kinase subdomains (SD) were determined based on the ABL kinase crystal structure (PDB ID 1IEP) [90] as the reference and in accordance to our previous study [123]: SDI:242-261(P-loop region); SD2:262-278; SD3:279-291(αC-helix); SD4:292-309; SD5:310-335 (hinge region); SD6A:336-356; SD6B357-374 (catalytic loop); SD7:375-393 (activation loop); SD8:394-416 (P+l loop); SD9:417-438; SD10:439-461; SD11:462-480; SD12:481-498. The C-terminal region encompasses SD8-SD12 subdomains.

We proposed that locally frustrated kinase sites may catalyze large scale cooperative transitions by activating specific pathways of allosteric transformation, which may be modulated by cancer mutations. According to this conjecture, structural localization of kinase cancer mutations would be collocated with the locally frustrated sites. Structural bioinformatics analysis of protein kinases has previously revealed that highly oncogenic kinase mutations could fall at structurally conserved positions within the kinase catalytic core [**123**]. Moreover, these structurally conserved mutational hotspots could be shared by multiple kinase genes. To test our hypotheses, we performed a comparative analysis of the spatial distribution of highly oncogenic kinase mutations and highly frustrated residues mapped onto the kinase catalytic domain **(** **Figure 6** **)**. We found that local frustration was not randomly scattered on the protein surface or uniformly distributed in the protein kinase structure. Interestingly, locally frustrated clusters could overlap with the kinase segments involved in allosteric interactions and collocate with the regions directly involved in conformational changes associated with the kinase function (**Figure 6**). In particular, our analysis revealed that the vast majority of locally frustrated sites resided in the C-terminal lobe, most notably populating the substrate binding region of the catalytic core framed by the αF, αG, αH, and αI helices, including the activation loop segment, and the P+1 loop (**Figure 6**). We have previously demonstrated that coupling between structurally rigid αF-helix (minimally frustrated site) and conformationally adaptive αI-helix, αC-helix and the P+1 loop (more frustrated sites) may control allosteric activation in protein kinases [**136**]. Our present results indicated that highly frustrated residues could be localized near hinges **(** **Figure 6** **)** coordinating collective motions of kinase regions during allosteric conformational changes. We also analyzed the distribution of known oncogenic mutations across catalytic core subdomains using a set of kinase oncogenes ABL, EGFR, BTK, KIT, BRAF, MET, and RET (**Figure 7A** **)**. It appeared that this distribution was characterized by a bias towards specific functional regions, and functionally important activation loop along with the downstream P+1 loop region tend to be more densely populated than other subdomains. Other segments such as P-loop and catalytic loop could also harbor oncogenic mutations, but were less frequently populated by functionally important mutations. Parallel with this analysis, we carried out structural mapping of highly frustrated residues onto the kinase catalytic core and quantified the distribution of the local frustration index as a function of the kinase subdomain **(** **Figure 7B** **)**. Importantly, the vast majority of highly frustrated kinase residues were mapped onto the C-terminal lobe, including the activation loop and regulatory P+1 loop. The locally frustrated residue clusters that populated the activation loop and the C-terminal lobe were collocated with the disease associated mutations and residues involved in allosteric conformational changes. Indeed, a relatively high concentration of highly frustrated residues in a single functional region is especially pronounced for the P+1 motif, which includes residues in the activation segment, and contains the conserved APE motif. The P+1 segment links the subdomains of the C-terminal lobe with the ATP and substrate binding regions in the N-terminal lobe. This segment is critical for protein substrate recognition and allosteric regulatory interactions [**75]**-[81****], serving as a hydrophobic glue holding the sub-domains of the C-lobe together. The APE motif is involved in allosteric regulation, as it is anchored to the αF, αG and αI-helices, providing direct communication between the activation segment and C-terminal. In addition, one of the highest concentrations of disease associated mutations localized in the vicinity of the P+1 pocket [**120]**-[123****]. Functional role of these residues as catalysts of kinase activation may be determined by their strategic location critical for regulation.

## Discussion

Activating kinase mutations result in a ligand-independent constitutive activation of the kinase activity. Among most prominent examples are activating mutations in the EGFR gene, where a single-point mutation L858R accounts for about 41% of all EGFR activating mutations [**140]**, [141****]. Strikingly, recent functional studies have revealed the impaired nuclear EGFR accumulation in cells expressing EGFR-L858R may be due to the lack of allosteric activation rather than a direct consequence of constitutive kinase activity [**142**]. Hence, the primary functional effect of the activating EGFR-L858R mutation, which was shown to thermodynamically stabilize EGFR [**134]**, [143****], is to allow for receptor activation that does not require the allosteric conformational change. The results of our current study corroborate with these central experimental findings. We observed a high density of locally frustrated residues in the regions involved in allosteric conformational transitions, particularly in the lower portion of the activation loop and the P+1 loop. While these locally frustrated sites could overlap with the mutational hotspots of disease-causing mutations, allosteric changes cannot occur if critical residues are mutated. This seeming contradiction may be partly explained based on the proposed functional role of locally frustrated sites as initiation points of allosteric transitions. Indeed, locally frustrated sites may trigger global structural changes via local rearrangements in the vicinity of pivotal hinge points and rigid body motions involving coupling of minimally frustrated and locally frustrated regions. The observed mutation-induced reduction of locally frustrated sites and thermodynamic stabilization of the active kinase form may thus help to suppress allosteric mechanism of activation.

This study suggested that the interplay between a minimally frustrated structural core and locally frustrated regions may collectively enable robust allosteric activation of protein kinases. Indeed, whereas a broad web of minimally frustrated residues in the kinase catalytic core could reflect robustness of the protein kinase fold to evolutionary pressure and mutations, the presence of locally frustrated residue clusters may not only be evolutionary tolerable but also potentially advantageous for tailoring protein kinase dynamics to maintain a dynamic equilibrium between alternative kinase states required for normal function. Diverse mechanisms of allosteric communication can span extreme cases, from a sequential model, where binding of a molecule at one site causes a sequential propagation of conformational changes across the protein to a fully cooperative model, where structural changes are tightly coupled and conformational switching is first-order phase transition. Our data seemed to support a “block-based” model of allosteric communication, according to which clusters of optimally interacting residues can recruit blocks of more flexible residues into communication pathways [**32**]. Although minimally frustrated residue clusters with optimized local interactions constitute the structurally rigid core of the kinase catalytic domain, locally frustrated residue clusters, whose interaction networks may not be energetically optimized, could define “soft spots”, that are weakly coupled to the kinase core and prone to dynamic modulation by mutations or binding.

In the present study, we combined computer simulations and the energy landscape analysis of protein kinases to characterize the interplay between oncogenic mutations, local frustration and protein flexibility as important catalysts of allostetric kinase activation and regulation. The results of this study suggested that mutation-induced allosteric signaling may involve a dynamic coupling between structurally rigid (minimally frustrated) and plastic (locally frustrated) clusters of residues. We found that the energy landscape effect of oncogenic mutations may be allosteric in nature, eliciting global changes in the spatial distribution of highly frustrated residues. Furthermore, the protein kinase regions undergoing large structural changes during allosteric transitions could be enriched in clusters of highly frustrated residues. The present study indicated that activating cancer mutations could act as catalysts of kinase activation by simultaneously perturbing the network of minimally frustrated interactions in the inactive kinase state, while reducing local frustration and allosterically restoring structural stability in the active kinase form. Allosterically induced switch in the state of locally frustrated residues upon mutation can shift the thermodynamic equilibrium and “lock” the oncogenic kinase in a constitutively active form. This may present a feasible mechanism by which oncogenic mutations may function as catalysts of kinase activation by detrimentally affecting the thermodynamic equilibrium between kinase states. The energy landsape analysis of protein kinases and the proposed role of locally frustrated sites in activation mechanisms may have useful implications for bioinformatics-based screening and detection of functional sites critical for allosteric regulation in complex biomolecular systems. The results may be also potentially interesting for protein design, where rationale engineering of locally frustrated regions may provide means for probing activation mechanisms in a desired regime.

## Materials and Methods

### Protein Kinase Mutants

Protein kinase sequences were obtained from Kinbase (http://kinase.com/kinbase/). Sequence analysis of protein kinase mutations was done using data collected from different sources, including PupaSNP [**144**], dbSNP database [**145**], Online Mendelian Inheritance in Man (OMIM) from National Center for Biotechnology Information (NCBI) [**146]**, [147****], KinMutBase [**148]**, [149****], BTKbase [**150**], Human gene mutation database (HGMD) [**151]**, [152****], Catalogue of Somatic Mutations in Cancer database (COSMIC) [**153**], Mutations of Kinases in Cancer (MoKCa) [**154**] SwissProt [**155]**-[157****] Protein Kinase Resource (PKR) [**158**], and PDB [**159**]. The assembled set of somatic kinase mutations was categorized based on a quantitative metric of oncogenic potential corresponding to the frequency profiles of somatic mutations in the protein kinases genes obtained from the COSMIC repository [**153**]. Since only a subset of cancer mutations can be directly mapped onto the crystal structure of the catalytic domain, there are some protein kinase genes with the known WT crystal structures, yet no mutational models could be reliably produced, because either all known mutations reside outside of the resolved crystal structure of the kinase catalytic domain or only synonymous mutations were available. A collection of somatic kinase mutations that corresponded to the catalytic domain included ABL (36 mutations), EGFR (85 mutations), BTK (100 mutations), KIT (54 mutations), BRAF (62 mutations), MET (46 mutations), and RET (39 mutations) (**Figure S8**). To facilitate structure-functional analysis, we generated structural models of various protein kinase mutants using the respective WT crystal structure as a template (see Supporting Information in **File S1**). A total of 57 kinase genes that covered a wide range of kinase subfamilies were used in the present study (**Table S1** in **File S1**).

### Analysis of Local Frustration in Protein Kinases

The protein kinase crystal structures as well as structural models of kinase mutants with the known WT crystal structure were used in the calculation of the residue-based configurational frustration index. We focused on the local frustration analysis conducted for ABL, EGFR, BTK, KIT, BRAF, MET, and RET kinase genes based on simulations of these kinases in both the inactive and active forms (see Supporting Information in **File S1**). These kinase genes also account for the vast majority of highly oncogenic mutations in the catalytic domain. We computed residue-based configurational frustration index via a web server (http://www.frustratometer.tk). The local frustration analysis adapted a recently proposed method of quantifying the degree of frustration manifested by spatially local protein interactions [**69**]. The local frustration index for the contact between the amino acids i,j was defined as a Z-score of the energy of the native pair compared to the N decoys. According to the Ferreiro-Wolynes model, a residue-based frustration index can measure the energetic stability of a particular native contact as compared to a set of all possible contacts sampled by automatic generation of ∼1000 distributed decoys and recomputing the energy change. The frustration index can be calculated by mutating the identities and the distances between the interacting amino acids. In the mutational frustration index, the decoy set randomizes only the identities of the interacting amino acids i, j while keeping all other interaction parameters at their native value. We employed a more general configurational frustration index, where the decoy set involved randomizing not only the residue identities but also the distance between the interacting amino acids i, j. The index value that corresponded to a positive Z-score value would indicate that the majority of other amino acid pairs in that position were unfavorable. A contact was defined as minimally frustrated if its native energy was at the lower end of the distribution of decoy energies, and a frustration index as measured by a Z-score would be of 0.78 or higher magnitude. Conversely, a contact was defined as highly frustrated if its native energy was at the higher end of the distribution with a local frustration index lower than -1. If the native energy was in between these limits, the contact was defined as neutral.

### Structural Modeling of Protein Kinases

The protein kinase crystal structures corresponding to 57 kinase genes were collected from PDB [**159**] and were employed in the structural bioinformatics analysis and biophysical modeling (**Table S1** in **File S1**). To facilitate structure-functional analysis of genetic variations in kinase genes, all crystal structures and mutational models were structurally aligned using a java- based multiple alignment tool STRAP (http://www.charite.de/bioinf/strap) and TM-align algorithm [**160**]. Structural modeling of kinase mutants was carried out using MODELLER [**161]**, [162****] with a subsequent refinement of side-chains by the SCRWL3 program [**163**]. Initial models were built in MODELLER using a flexible sphere of 5 Å around mutated residue. A protocol involving a conjugate gradient (CG) minimization, followed by simulated annealing refinement was repeated 20 times to generate 100 initial models for each studied mutant. The mutational models were chosen out of the 100 models as scored by the MODELLER default scoring function, followed by structural refinement using MD simulations protocol detailed in [**123**]. MD refinement simulations were done using NAMD 2.6 [**164**] with the CHARMM27 force field [**165]**, [166****] and the explicit TIP3P water model as implemented in NAMD 2.6 [**167**]. The VMD program was used for the preparation and analysis of simulations [**168]**, [169****]. Protein kinase flexibility was also analyzed by combining the results of MD simulations with the principal component analysis of conformational ensembles [**170]**, [171****].

## Supporting Information

Figure S1 **The Effect of Oncogenic Mutations on Local Frustration in the Inhibitor-bound ABL Kinase Structures.** The residue-based frustration index values are shown for a set of oncogenic ABL kinase mutants in the inactive (A) and active forms (B). The frustration index values are shown in filled yellow bars for the WT kinase form and in red filled bars for the mutant forms. The analysis was performed using inhibitor-bound crystal structures of ABL in the inactive form (PDB ID 1IEP) [**90**] and active form (PDB ID 1M52) [**91]**, [92****].
(TIF)

Figure S2 **The Effect of Oncogenic Mutations on Local Frustration in the Inhibitor-bound EGFR Kinase Structures.** The residue-based frustration index values are shown for a set of oncogenic EGFR kinase mutants in the inactive (A) and active forms (B). The frustration index values are shown in filled yellow bars for the WT kinase form and in red filled bars for the mutant forms. The analysis was performed using inhibitor-bound crystal structures of EGFR in the inactive form (PDB ID 1XKK) [**117**] and active form (PDB ID 2J6M) [**118**].
(TIF)

Figure S3 **The Energy Landscape of the ABL Kinase Catalytic Domain.** The spatial distribution and partition of minimally frustrated and locally frustrated regions in the inactive ABL kinase (**A**) and active ABL state (**B**). The protein backbone is displayed as blue ribbons, the direct residue interactions are shown with solid lines. Minimally frustrated interactions are shown in green, highly frustrated contacts in red, neutral contacts are not drawn. This analysis illustrated common similarities and differences in the local frustration of inactive and active kinase forms. Structural mapping of local frustration on the ABL kinase catalytic core is shown for the inactive ABL-WT structure (PDB ID 1IEP) [**90**] and active ABL-WT structure (PDB ID 1M52) [**91]**, [92****]. The VMD program was used for protein kinase structure visualization [**168]**, [169****].
(TIF)

Figure S4 **A Residue-based Comparative Analysis of Local Frustration and Protein Flexibility.** The values of the B-factors (A), the configurational frustration index FI (B), and the RMSF (C) for the protein kinase residues. The crystal structure of EGFR-WT in the active form (PDB ID 2J6M) [**118**] was used in this example of a comparative analysis.
(TIF)

Figure S5 **Structural Mapping of B-factors and Locally Frustrated Sites onto the Kinase Catalytic Core.** Structural mapping of the average B-factors and locally frustrated residues onto a set of inactive (**A**) and active kinase structures (**B**). The set of inactive kinase conformations included ABL (PDB 1IEP), KIT (PDB ID 1T45), MET (PDB ID 2G15) and BRAF (PDB ID 1UWH). The set of active kinase conformations included EGFR (PDB ID 2J6M), BTK (PDB ID 1K2P), and RET (PDB ID 2IVS). The protein residues were colored accordingly to their B-factor (temperature factor) from dark blue for low B-factor to red for high B-factor. The locally frustrated residues are shown as red spheres. The Pymol program was used for visualization of protein kinase structures and the local frustration mapping (The PyMOL Molecular Graphics System, Version 1.2r3pre, Schrödinger, and LLC.).
(TIF)

Figure S6 **Mutation-induced Redistribution of the Local Frustration in the ABL Kinase DFG Motif.** The mutation-induced changes in the local frustration between ABL-WT (A) and ABL-T315I (B).
(TIF)

Figure S7 **Mutation-induced Redistribution of the Local Frustration in the ABL Kinase Activation Loop.** The mutation-induced changes in the local frustration between ABL-WT (A) and ABL-T315I (B).
(TIF)

Figure S8 **Gene-based Pie Diagram of Kinase Cancer Mutations.** The arc length of each sector is proportional to the number of cancer mutations for a given kinase gene. For clarity of presentation, only top 70 kinase genes with the cancer-causing mutations that can be mapped onto three-dimensional structure of the catalytic core are presented.
(TIF)

File S1 **Supporting tables.**
(DOC)
