# Supplementary figures and images for: The Energy Landscape Analysis of Cancer Mutations in Protein Kinases
Source: PLoS One. 2011 Oct 6;6(10):e26071. doi: 10.1371/journal.pone.0026071 (PMC3188581; doi:10.1371/journal.pone.0026071)

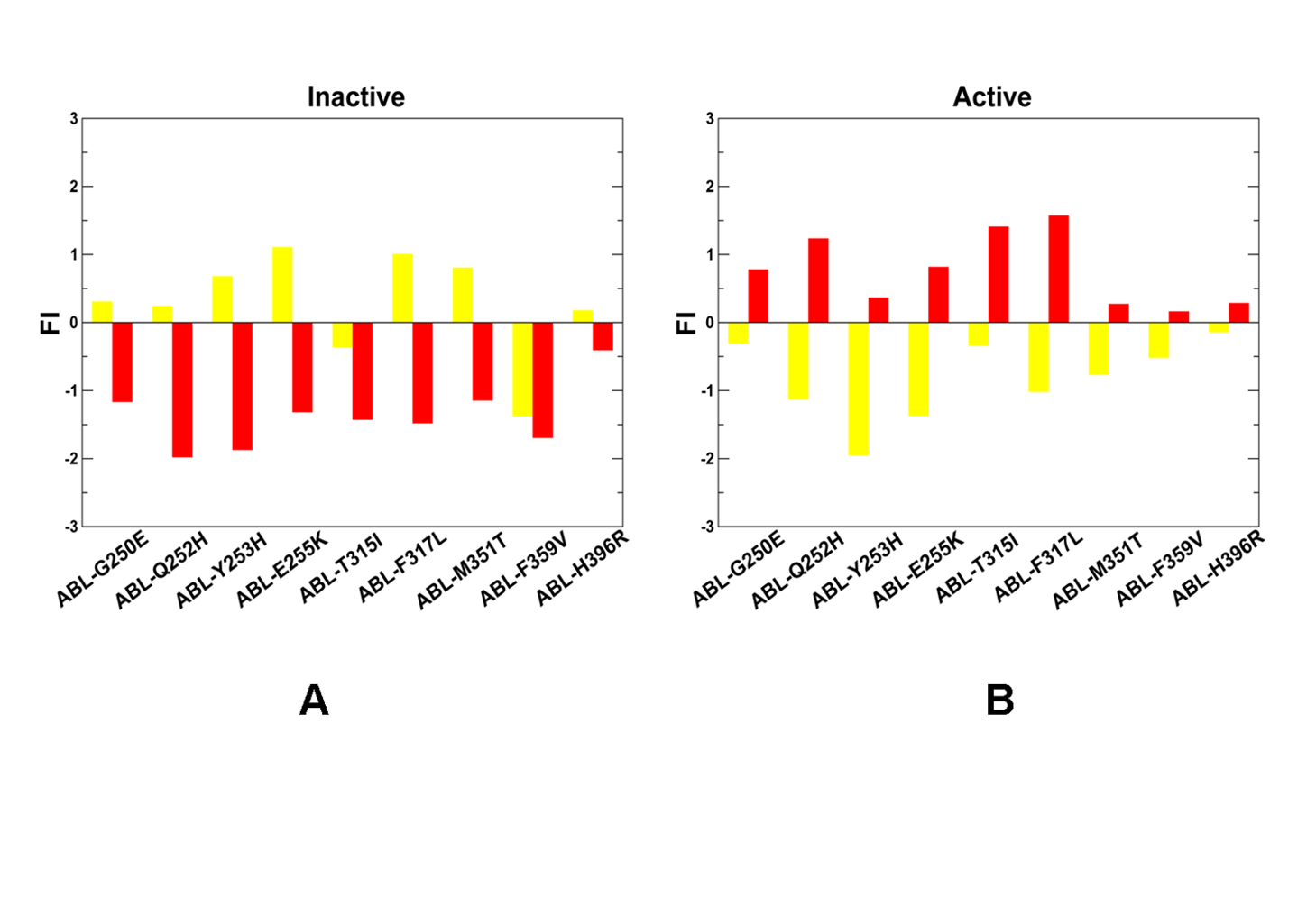

Supplement: Figure S1 — The Effect of Oncogenic Mutations on Local Frustration in the Inhibitor-bound ABL Kinase Structures. The residue-based frustration index values are shown for a set of oncogenic ABL kinase mutants in the inactive (A) and active forms (B). The frustration index values are shown in filled yellow bars for the WT kinase form and in red filled bars for the mutant forms. The analysis was performed using inhibitor-bound crystal structures of ABL in the inactive form (PDB ID 1IEP) [90] and active form (PDB ID 1M52) [91] , [92]. (TIF) [file pone.0026071.s001.tif]

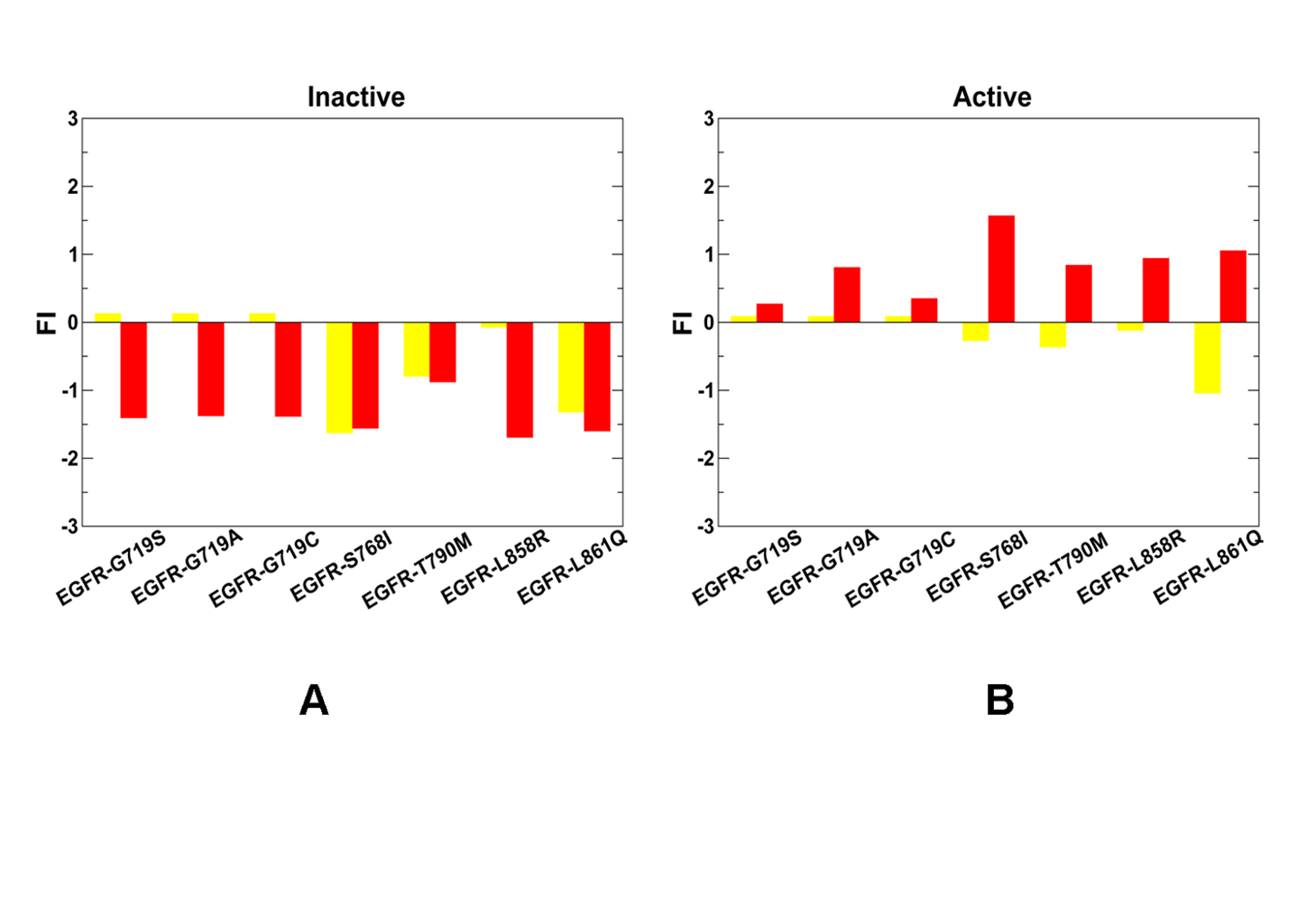

Supplement: Figure S2 — The Effect of Oncogenic Mutations on Local Frustration in the Inhibitor-bound EGFR Kinase Structures. The residue-based frustration index values are shown for a set of oncogenic EGFR kinase mutants in the inactive (A) and active forms (B). The frustration index values are shown in filled yellow bars for the WT kinase form and in red filled bars for the mutant forms. The analysis was performed using inhibitor-bound crystal structures of EGFR in the inactive form (PDB ID 1XKK) [117] and active form (PDB ID 2J6M) [118]. (TIF) [file pone.0026071.s002.tif]

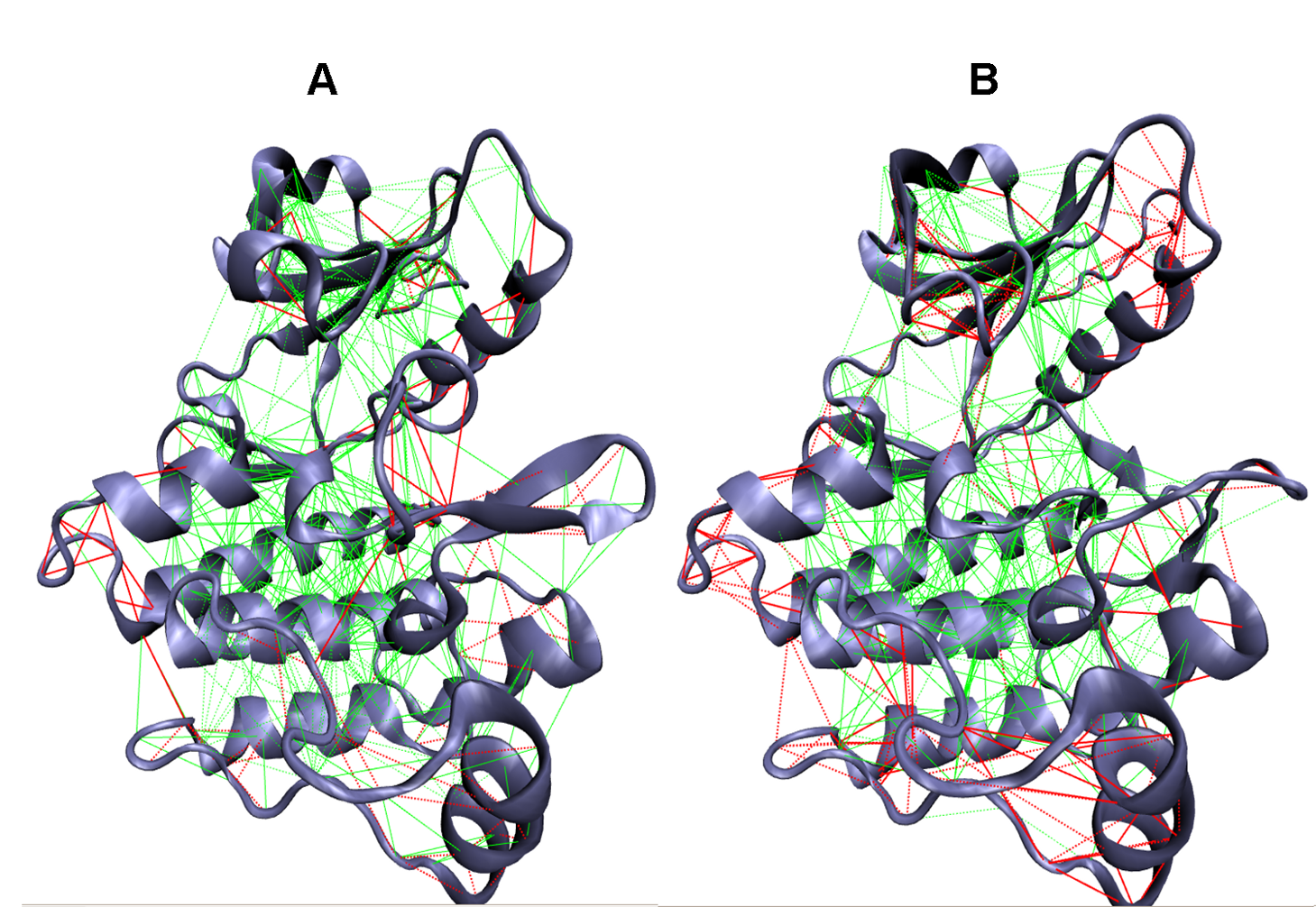

Supplement: Figure S3 — The Energy Landscape of the ABL Kinase Catalytic Domain. The spatial distribution and partition of minimally frustrated and locally frustrated regions in the inactive ABL kinase (A) and active ABL state (B). The protein backbone is displayed as blue ribbons, the direct residue interactions are shown with solid lines. Minimally frustrated interactions are shown in green, highly frustrated contacts in red, neutral contacts are not drawn. This analysis illustrated common similarities and differences in the local frustration of inactive and active kinase forms. Structural mapping of local frustration on the ABL kinase catalytic core is shown for the inactive ABL-WT structure (PDB ID 1IEP) [90] and active ABL-WT structure (PDB ID 1M52) [91] , [92]. The VMD program was used for protein kinase structure visualization [168] , [169]. (TIF) [file pone.0026071.s003.tif]

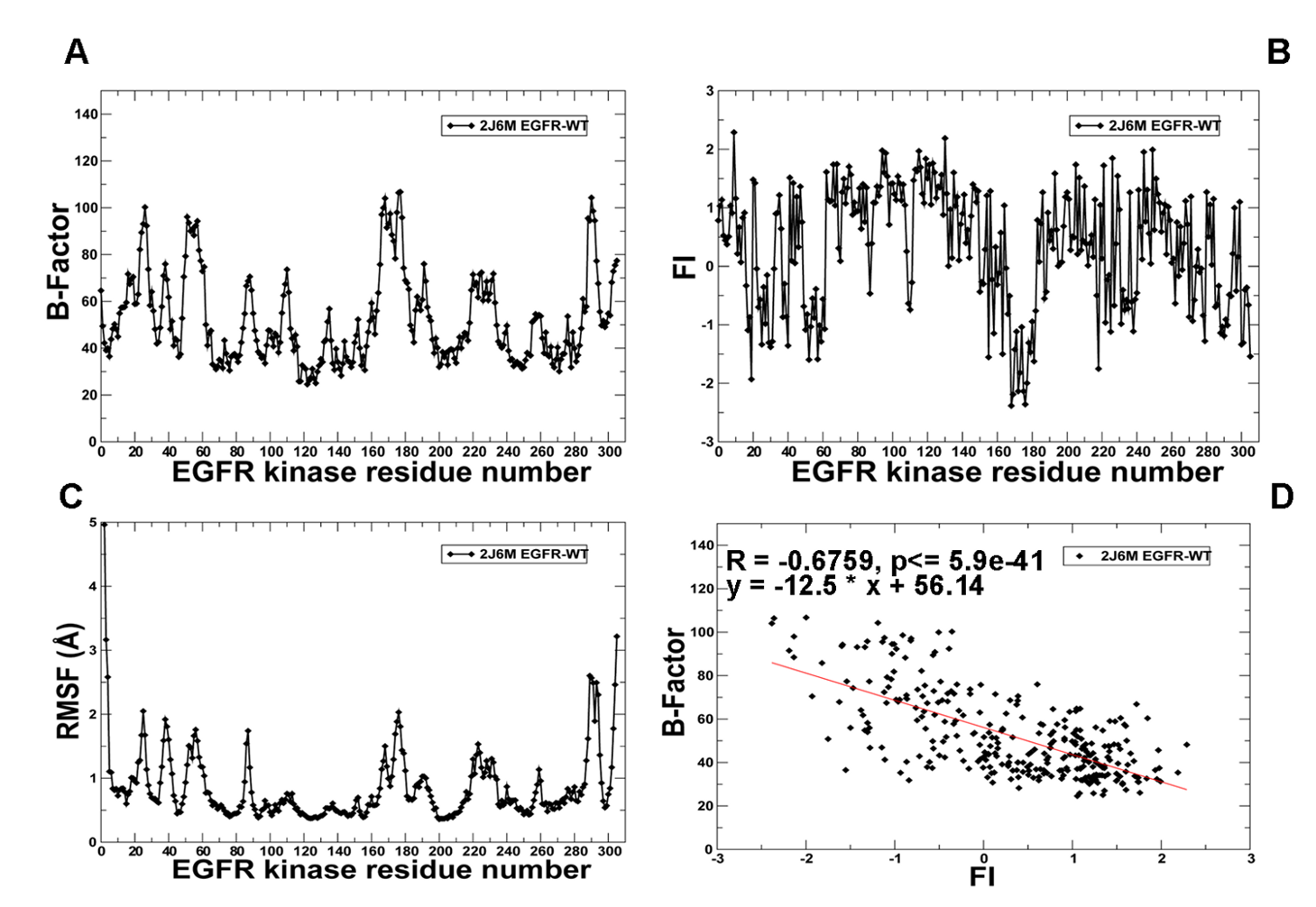

Supplement: Figure S4 — A Residue-based Comparative Analysis of Local Frustration and Protein Flexibility. The values of the B-factors (A), the configurational frustration index FI (B), and the RMSF (C) for the protein kinase residues. The crystal structure of EGFR-WT in the active form (PDB ID 2J6M) [118] was used in this example of a comparative analysis. (TIF) [file pone.0026071.s004.tif]

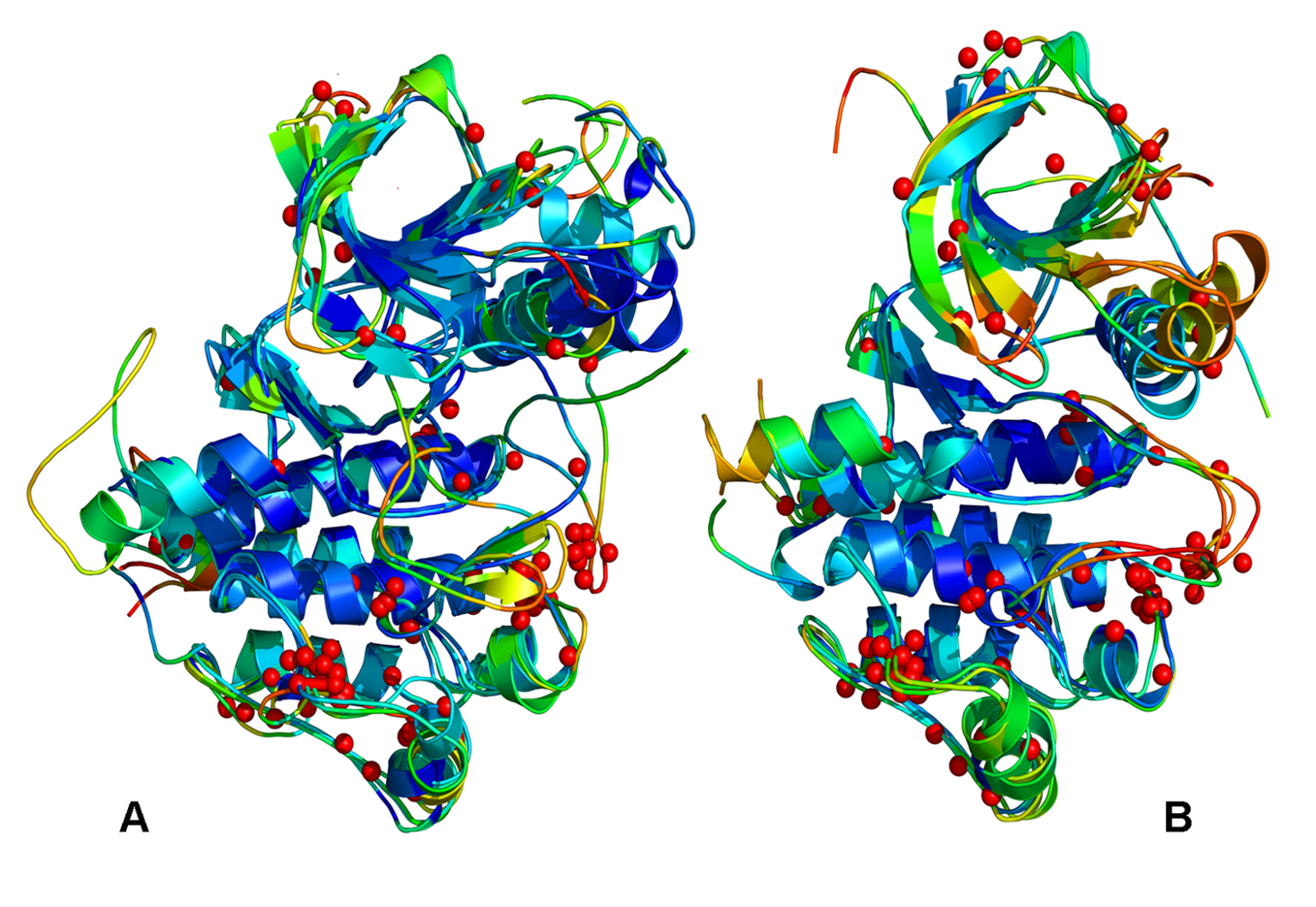

Supplement: Figure S5 — Structural Mapping of B-factors and Locally Frustrated Sites onto the Kinase Catalytic Core. Structural mapping of the average B-factors and locally frustrated residues onto a set of inactive (A) and active kinase structures (B). The set of inactive kinase conformations included ABL (PDB 1IEP), KIT (PDB ID 1T45), MET (PDB ID 2G15) and BRAF (PDB ID 1UWH). The set of active kinase conformations included EGFR (PDB ID 2J6M), BTK (PDB ID 1K2P), and RET (PDB ID 2IVS). The protein residues were colored accordingly to their B-factor (temperature factor) from dark blue for low B-factor to red for high B-factor. The locally frustrated residues are shown as red spheres. The Pymol program was used for visualization of protein kinase structures and the local frustration mapping (The PyMOL Molecular Graphics System, Version 1.2r3pre, Schrödinger, and LLC.). (TIF) [file pone.0026071.s005.tif]

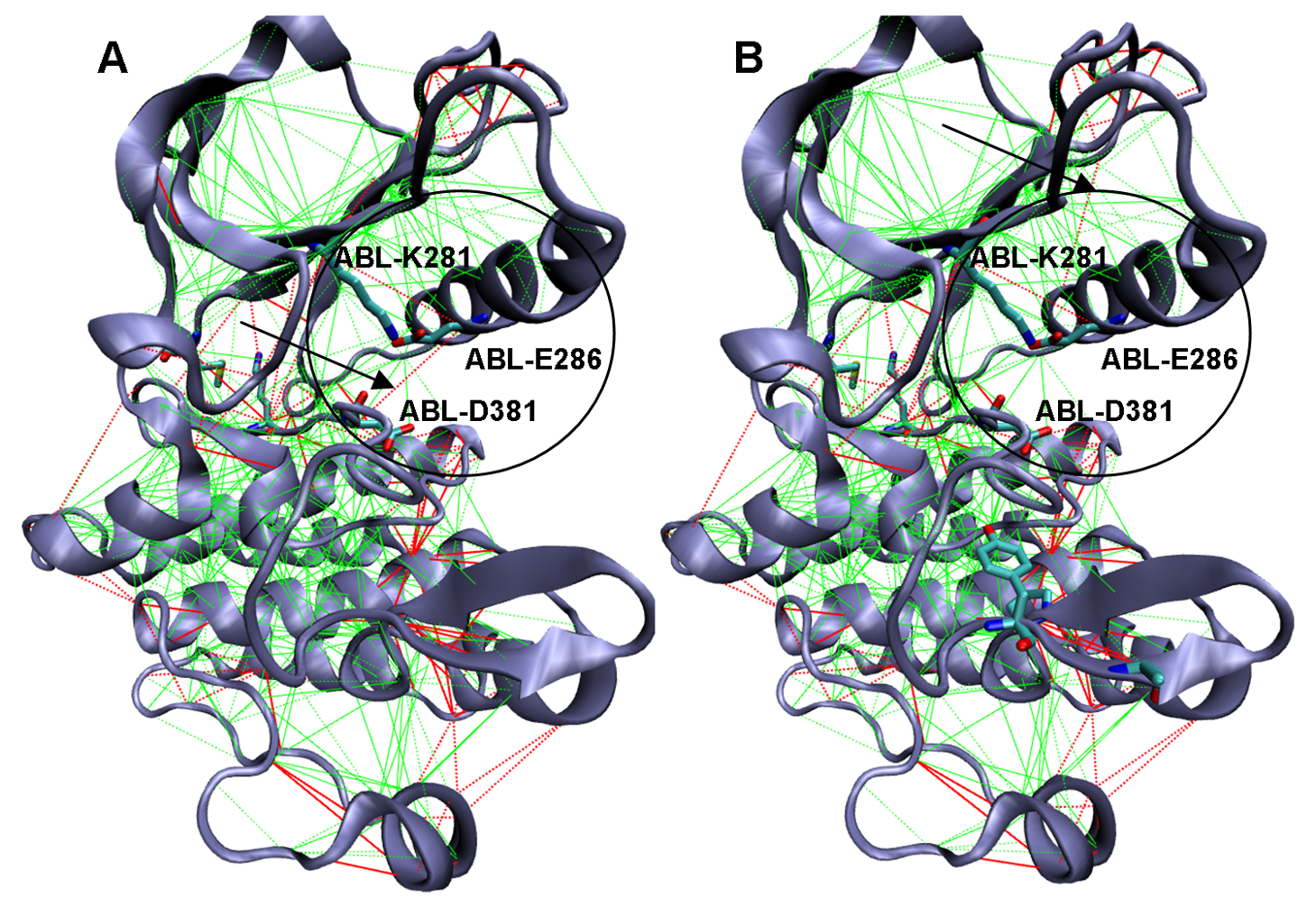

Supplement: Figure S6 — Mutation-induced Redistribution of the Local Frustration in the ABL Kinase DFG Motif. The mutation-induced changes in the local frustration between ABL-WT (A) and ABL-T315I (B). (TIF) [file pone.0026071.s006.tif]

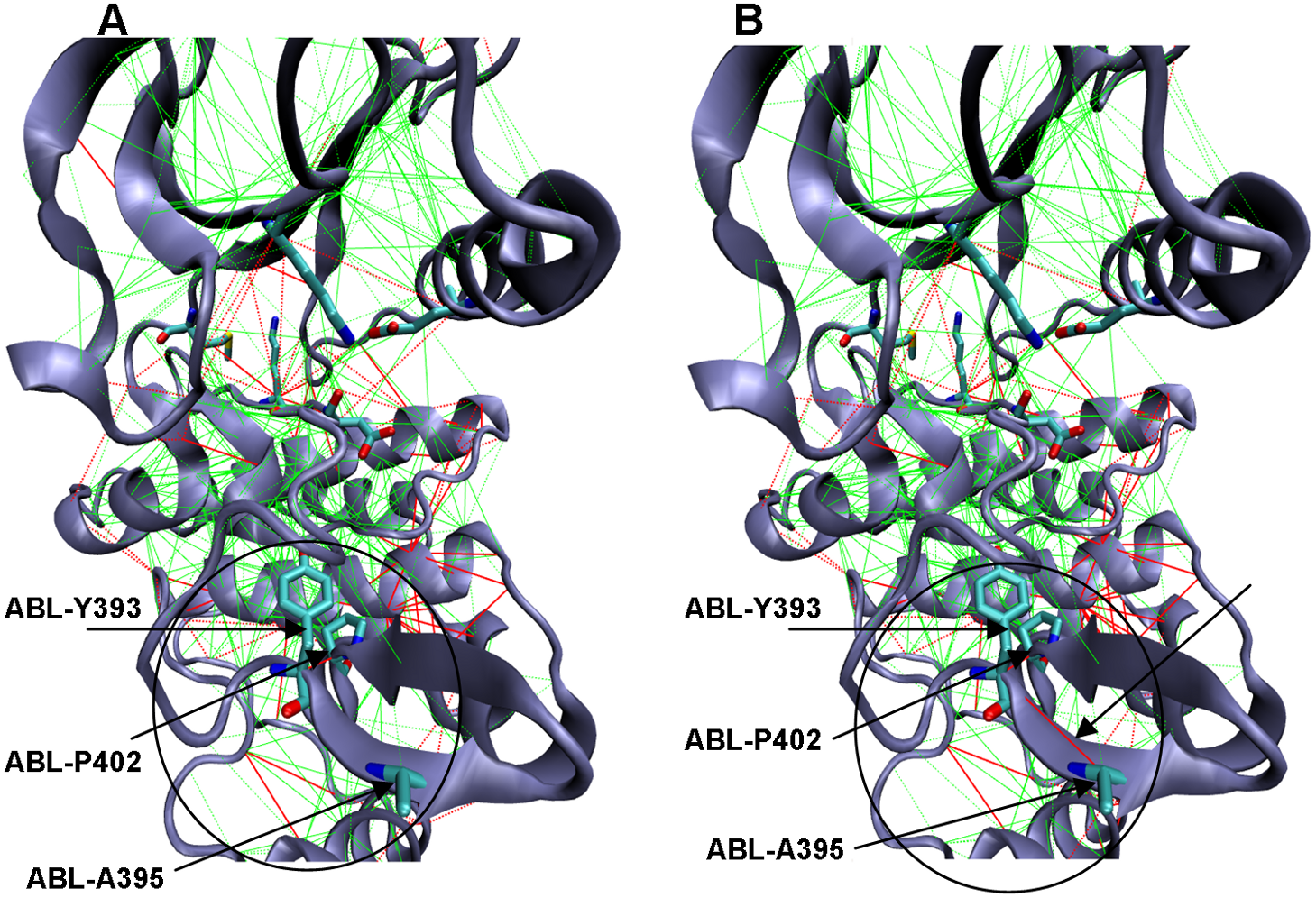

Supplement: Figure S7 — Mutation-induced Redistribution of the Local Frustration in the ABL Kinase Activation Loop. The mutation-induced changes in the local frustration between ABL-WT (A) and ABL-T315I (B). (TIF) [file pone.0026071.s007.tif]

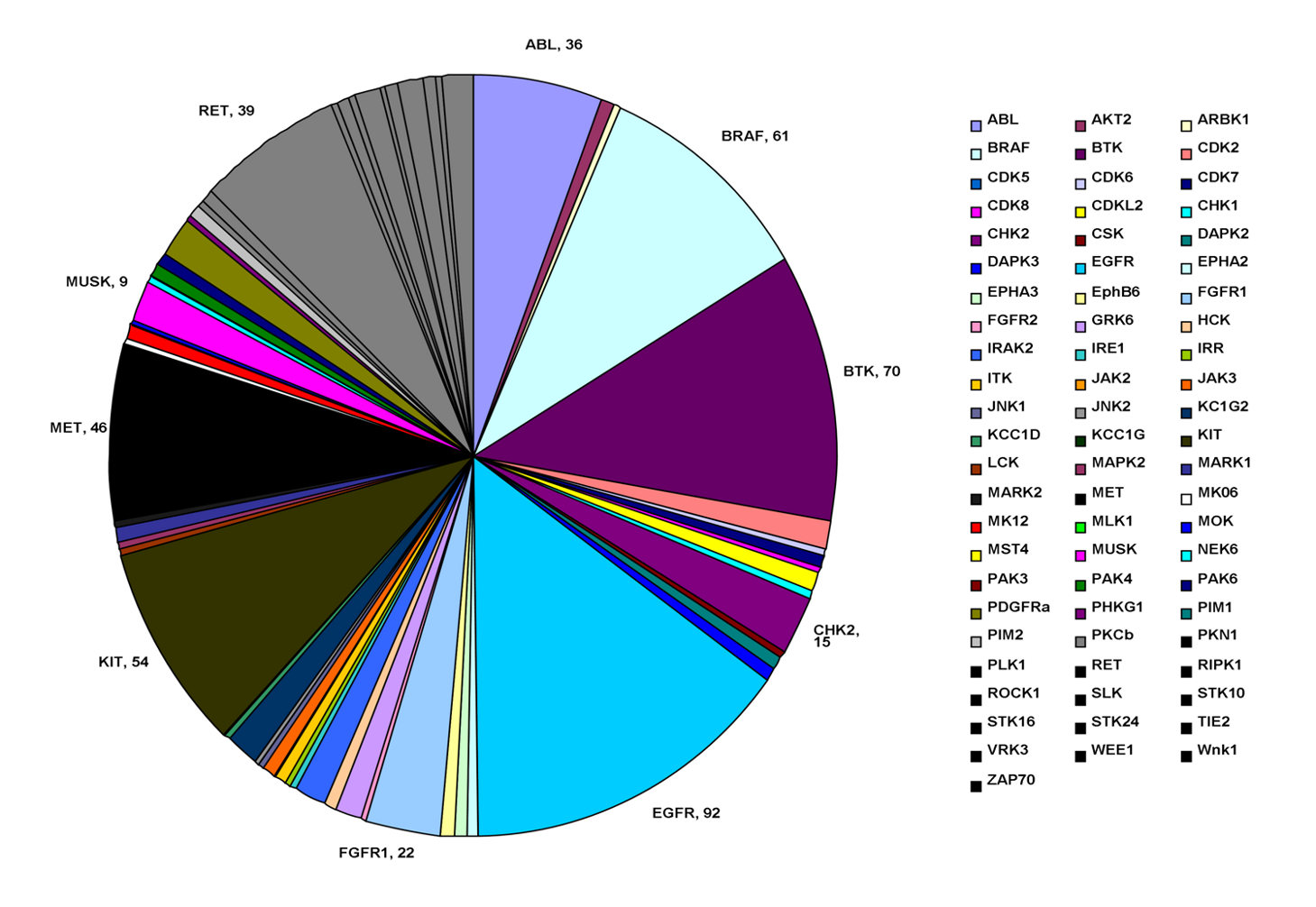

Supplement: Figure S8 — Gene-based Pie Diagram of Kinase Cancer Mutations. The arc length of each sector is proportional to the number of cancer mutations for a given kinase gene. For clarity of presentation, only top 70 kinase genes with the cancer-causing mutations that can be mapped onto three-dimensional structure of the catalytic core are presented. (TIF) [file pone.0026071.s008.tif]
