# Supplementary material for: The Energy Landscape Analysis of Cancer Mutations in Protein Kinases
Source: PLoS One. 2011 Oct 6;6(10):e26071. doi: 10.1371/journal.pone.0026071 (PMC3188581; doi:10.1371/journal.pone.0026071)
Supplement: File S1 — Supporting tables. (DOC) [file pone.0026071.s009.doc]

**Table S1. The crystal structures and kinase genes employed in structural modeling of cancer mutants and the energy landscape analysis.***

**Kinase Gene Wild-type crystal structure (PDB ID) # Modeled Mutations**

**ABL 1IEP/1M52 36**

**AKT2 1GZK 5**

**ARBK1 1OMW 5**

**BRAF 1UWH/3C4D 62**

**BTK 1K2P/ 100**

**CDK2 1FVV 6**

**CDK5 1UNG 1**

**CDK6 1BLX 2**

**CDK7 1UA2 3**

**CHK1 1IA8 2**

**CSK 1BYG 3**

**DAPK2 2A2A 3**

**DAPK3 1YRP 3**

**EGFR 1XKK/2J6M 85**

**EPHA2 1MQB 3**

**EPHA3 2QO9 4**

**FGFR1 1FGK 22**

**GRK6 2ACX 7**

**HCK 1QCF 4**

**IRE1 2B3Y 3**

**ITK 1SM2 3**

**JNK1 2H96 2**

**JNK2 2H96 1**

**KC1G2 2C47 9**

**KC1G3 2CHL 1**

**KCC1D 2JC6 1**

**KCC1G 2JAM 1**

**KIT 1T45/1PKG 54**

**LCK 1QPE 3**

**LOK 2J7T 1**

**MAPK2 1KWP 2**

**MARK2 1ZMU 3**

**MET 2G15 46**

**ERK3 2C30 1**

**MK12/P38 1CM8 3**

**MLK1 1IAS 1**

**MP2K2 1S9I 1**

**MST4 2VX3 1**

**PAK1 1YHV 1**

**PAK4 2BVA 3**

**PAK6 2C30 3**

**PAK7 2F57 1**

**PIM1 1XQZ 1**

**PIM2 2IWI 3**

**PKCB 2I0E 1**

**PLK1 2OU7 3**

**RET 1XPD/2IVS 39**

**ROCK1 2ETR 2**

**SLK 2J51 1**

**STK16 2BUJ 7**

**STK6 1MQ4 1**

**TGFR1 1B6C 1**

**TIE2 3ALN 4**

**VRK3 2JII 7**

**WEE1 1X8B 3**

**WNK1 1T4H 1**

**ZAP70 1U59 9**

* These kinase genes have the known crystal structure of the wild type catalytic domain. Structural models of kinase mutants were generated using the wild type crystal structure as a template.

**Table S2. The protein kinase crystal structures and structural models of highly oncogenic kinase mutants used in the energy landscape analysis.**

**Kinase Structure (PDB ID_Mutational Status) Modeled State**

**ABL 1IEP_WT Inactive**

**ABL 1FPU_WT Inactive**

**ABL 1OPJ_WT Inactive**

**ABL 1M52_WT Active**

**ABL 1OPK_WT Active**

**ABL 1OPL_WT Active**

**ABL 2G1T_WT Inactive**

**ABL 2Z60_T315I Active**

**ABL 2V7A_T315I Active**

**ABL 2QOH_T315I Active**

**ABL 3DK7_T315I Active**

**ABL 3IK3_T315I Active**

**ABL 3OY3_T315I Active**

**ABL 3QRJ_T315I Inactive**

**ABL D276G Inactive/Active**

**ABL E255K Inactive/Active**

**ABL E255V Inactive/Active**

**ABL E352G Inactive/Active**

**ABL E355G Inactive/Active**

**ABL E373G Inactive/Active**

**ABL E459K Inactive/Active**

**ABL F311L Inactive/Active**

**ABL F317L Inactive/Active**

**ABL F359A Inactive/Active**

**ABL F359V Inactive/Active**

**ABL F382L Inactive/Active**

**ABL F486S Inactive/Active**

**ABL G250E Inactive/Active**

**ABL G321E Inactive/Active**

**ABL H396P Inactive/Active**

**ABL H396R Inactive/Active**

**ABL K247R Inactive/Active**

**ABL L248V Inactive/Active**

**ABL L387M Inactive/Active**

**ABL M244V Inactive/Active**

**ABL M343T Inactive/Active**

**ABL M351T Inactive/Active**

**ABL Q252E Inactive/Active**

**ABL Q252H Inactive/Active**

**ABL Q252R Inactive/Active**

**ABL S417Y Inactive/Active**

**ABL T315N Inactive/Active**

**ABL T389A Inactive/Active**

**ABL V304G Inactive/Active**

**ABL V371A Inactive/Active**

**ABL V379I Inactive/Active**

**ABL Y253F Inactive/Active**

**ABL Y253H Inactive/Active**

**ABL Y353H Inactive/Active**

**EGFR 1XKK_WT Inactive**

**EGFR 2GS7_WT Inactive**

**EGFR 1M14_WT Active
EGFR 1M17_WT Active**

**EGFR 2GS2_WT Active**

**EGFR 2GS6_WT Active**

**EGFR 2J6M_WT Active**

**EGFR 2ITX_WT Active**

**EGFR 2ITY_WT Active**

**EGFR 2ITW_WT Active**

**EGFR 2JIT_T790M Active**

**EGFR 2JIU_T790M Active**

**EGFR 2JIV_T790M Active**

**EGFR 2ITN_G719S Active**

**EGFR 2ITO_G719S Active**

**EGFR 2ITP_G719S Active**

**EGFR 2ITQ_G719S Active**

**EGFR 2ITT_L858R Active**

**EGFR 2ITU_L858R Active**

**EGFR 2ITV_L858R Active**

**EGFR 2ITZ_L858R Active**

**EGFR A743P Inactive/Active**

**EGFR A743S Inactive/Active**

**EGFR A750P Inactive/Active**

**EGFR A839T Inactive/Active**

**EGFR A859T Inactive/Active**

**EGFR A864T Inactive/Active**

**EGFR D761N Inactive/Active**

**EGFR D761Y Inactive/Active**

**EGFR D770N Inactive/Active**

**EGFR E734K Inactive/Active**

**EGFR E746K Inactive/Active**

**EGFR E746V Inactive/Active**

**EGFR E804G Inactive/Active**

**EGFR E866K Inactive/Active**

**EGFR F856L Inactive/Active**

**EGFR G719A Inactive/Active**

**EGFR G719C Inactive/Active**

**EGFR G719D Inactive/Active**

**EGFR G724S Inactive/Active**

**EGFR G729E Inactive/Active**

**EGFR G735S Inactive/Active**

**EGFR G779F Inactive/Active**

**EGFR G796S Inactive/Active**

**EGFR G810S Inactive/Active**

**EGFR G863D Inactive/Active**

**EGFR G873E Inactive/Active**

**EGFR H773L Inactive/Active**

**EGFR H773R Inactive/Active**

**EGFR H835L Inactive/Active**

**EGFR H850N Inactive/Active**

**EGFR I715S Inactive/Active**

**EGFR I853T Inactive/Active**

**EGFR K745R Inactive/Active**

**EGFR K754R Inactive/Active**

**EGFR K846R Inactive/Active**

**EGFR L718P Inactive/Active**

**EGFR L730F Inactive/Active**

**EGFR L792P Inactive/Active**

**EGFR L798F Inactive/Active**

**EGFR L833V Inactive/Active**

**EGFR L838V Inactive/Active**

**EGFR L858A Inactive/Active**

**EGFR L858M Inactive/Active**

**EGFR L858W Inactive/Active**

**EGFR L861Q Inactive/Active**

**EGFR L861R Inactive/Active**

**EGFR N826S Inactive/Active**

**EGFR P733L Inactive/Active**

**EGFR P733S Inactive/Active**

**EGFR P741L Inactive/Active**

**EGFR P753F Inactive/Active**

**EGFR P753S Inactive/Active**

**EGFR Q787R Inactive/Active**

**EGFR Q812R Inactive/Active**

**EGFR R776C Inactive/Active**

**EGFR R776H Inactive/Active**

**EGFR R803L Inactive/Active**

**EGFR R832H Inactive/Active**

**EGFR R841K Inactive/Active**

**EGFR S720F Inactive/Active**

**EGFR S752Y Inactive/Active**

**EGFR S768C Inactive/Active**

**EGFR S768I Inactive/Active**

**EGFR S784F Inactive/Active**

**EGFR T725M Inactive/Active**

**EGFR T751I Inactive/Active**

**EGFR T783I Inactive/Active**

**EGFR T847I Inactive/Active**

**EGFR V742A Inactive/Active**

**EGFR V769L Inactive/Active**

**EGFR V769M Inactive/Active**

**EGFR V774M Inactive/Active**

**EGFR V802I Inactive/Active**

**EGFR V819A Inactive/Active**

**EGFR V834L Inactive/Active**

**EGFR V834M Inactive/Active**

**EGFR V843I Inactive/Active**

**EGFR V851A Inactive/Active**

**EGFR V851I Inactive/Active**

**EGFR V897I Inactive/Active**

**EGFR W731R Inactive/Active**

**EGFR Y727C Inactive/Active**

**BRAF 1UWH_WT Inactive**

**BRAF 1UWJ_V599E Inactive**

**BRAF 3C4D_V600E Active**

**BRAF 3IDP_V600E Active**

**BRAF 3OG7_V600E Active**

**BRAF A598V Inactive/Active**

**BRAF D587A Inactive/Active**

**BRAF D587E Inactive/Active**

**BRAF D594E Inactive/Active**

**BRAF D594G Inactive/Active**

**BRAF D594K Inactive/Active**

**BRAF D594V Inactive/Active**

**BRAF E586K Inactive/Active**

**BRAF F468C Inactive/Active**

**BRAF F595L Inactive/Active**

**BRAF F595S Inactive/Active**

**BRAF G464E Inactive/Active**

**BRAF G464R Inactive/Active**

**BRAF G464V Inactive/Active**

**BRAF G466A Inactive/Active**

**BRAF G466E Inactive/Active**

**BRAF G466R Inactive/Active**

**BRAF G466V Inactive/Active**

**BRAF G469A Inactive/Active**

**BRAF G469E Inactive/Active**

**BRAF G469R Inactive/Active**

**BRAF G469S Inactive/Active**

**BRAF G469V Inactive/Active**

**BRAF G596R Inactive/Active**

**BRAF G606E Inactive/Active**

**BRAF G615R Inactive/Active**

**BRAF H608R Inactive/Active**

**BRAF I463S Inactive/Active**

**BRAF I582M Inactive/Active**

**BRAF I592M Inactive/Active**

**BRAF I592V Inactive/Active**

**BRAF K475M Inactive/Active**

**BRAF K601E Inactive/Active**

**BRAF K601N Inactive/Active**

**BRAF L588R Inactive/Active**

**BRAF L597Q Inactive/Active**

**BRAF L597R Inactive/Active**

**BRAF L597S Inactive/Active**

**BRAF L597V Inactive/Active**

**BRAF L618S Inactive/Active**

**BRAF N581S Inactive/Active**

**BRAF R462I Inactive/Active**

**BRAF S605F Inactive/Active**

**BRAF S605G Inactive/Active**

**BRAF S605N Inactive/Active**

**BRAF S607P Inactive/Active**

**BRAF S614P Inactive/Active**

**BRAF S616P Inactive/Active**

**BRAF T589I Inactive/Active**

**BRAF T599I Inactive/Active**

**BRAF V459L Inactive/Active**

**BRAF V600A Inactive/Active**

**BRAF V600D Inactive/Active**

**BRAF V600G Inactive/Active**

**BRAF V600K Inactive/Active**

**BRAF V600L Inactive/Active**

**BRAF V600M Inactive/Active**

**BRAF V600R Inactive/Active**

**BRAF W604G Inactive/Active**

**BRAF W619R Inactive/Active**

**BTK 1K2P_WT Inactive**

**BTK 3K54_WT Inactive/Active**

**BTK 3GEN_WT Active**

**BTK 3PIX_WT Inactive/Active**

**BTK 3PIY_WT Inactive/Active**

**BTK 3PIZ_WT Inactive/Active**

**BTK 3PJ1_WT Active**

**BTK 3PJ2_WT Active**

**BTK 3PJ3_WT Active**

**BTK 3OCT_V555R Active**

**BTK A508D Inactive/Active**

**BTK A523E Inactive/Active**

**BTK A582V Inactive/Active**

**BTK A607D Inactive/Active**

**BTK A622P Inactive/Active**

**BTK C502F Inactive/Active**

**BTK C502W Inactive/Active**

**BTK C506R Inactive/Active**

**BTK C506Y Inactive/Active**

**BTK C527S Inactive/Active**

**BTK C633Y Inactive/Active**

**BTK D504V Inactive/Active**

**BTK D521G Inactive/Active**

**BTK D521H Inactive/Active**

**BTK D521N Inactive/Active**

**BTK E445D Inactive/Active**

**BTK E567K Inactive/Active**

**BTK E589D Inactive/Active**

**BTK E589G Inactive/Active**

**BTK E589K Inactive/Active**

**BTK F559S Inactive/Active**

**BTK F583S Inactive/Active**

**BTK F644L Inactive/Active**

**BTK F644S Inactive/Active**

**BTK G414R Inactive/Active**

**BTK G462D Inactive/Active**

**BTK G462V Inactive/Active**

**BTK G541D Inactive/Active**

**BTK G584W Inactive/Active**

**BTK G594E Inactive/Active**

**BTK G594R Inactive/Active**

**BTK G613D Inactive/Active**

**BTK H454R Inactive/Active**

**BTK I429N Inactive/Active**

**BTK K430E Inactive/Active**

**BTK K430R Inactive/Active**

**BTK L408P Inactive/Active**

**BTK L452P Inactive/Active**

**BTK L486P Inactive/Active**

**BTK L498V Inactive/Active**

**BTK L512P Inactive/Active**

**BTK L512Q Inactive/Active**

**BTK L518R Inactive/Active**

**BTK L542P Inactive/Active**

**BTK L569P Inactive/Active**

**BTK L616F Inactive/Active**

**BTK L647P Inactive/Active**

**BTK L647R Inactive/Active**

**BTK L648P Inactive/Active**

**BTK L652P Inactive/Active**

**BTK M477R Inactive/Active**

**BTK M509I Inactive/Active**

**BTK M509V Inactive/Active**

**BTK M587L Inactive/Active**

**BTK M630I Inactive/Active**

**BTK M630K Inactive/Active**

**BTK M630T Inactive/Active**

**BTK N526K Inactive/Active**

**BTK P565T Inactive/Active**

**BTK P566S Inactive/Active**

**BTK P597T Inactive/Active**

**BTK P619A Inactive/Active**

**BTK P619S Inactive/Active**

**BTK P619T Inactive/Active**

**BTK P642L Inactive/Active**

**BTK Q612P Inactive/Active**

**BTK R520Q Inactive/Active**

**BTK R525G Inactive/Active**

**BTK R525P Inactive/Active**

**BTK R525Q Inactive/Active**

**BTK R544G Inactive/Active**

**BTK R544K Inactive/Active**

**BTK R562P Inactive/Active**

**BTK R562W Inactive/Active**

**BTK R615P Inactive/Active**

**BTK R618G Inactive/Active**

**BTK R641C Inactive/Active**

**BTK R641H Inactive/Active**

**BTK S538P Inactive/Active**

**BTK S575R Inactive/Active**

**BTK S578Y Inactive/Active**

**BTK S592P Inactive/Active**

**BTK S623L Inactive/Active**

**BTK T606P Inactive/Active**

**BTK T628A Inactive/Active**

**BTK T643I Inactive/Active**

**BTK V535F Inactive/Active**

**BTK V537E Inactive/Active**

**BTK V585F Inactive/Active**

**BTK V626G Inactive/Active**

**BTK W563L Inactive/Active**

**BTK W581R Inactive/Active**

**BTK W588C Inactive/Active**

**BTK W634S Inactive/Active**

**BTK Y418H Inactive/Active**

**BTK Y476D Inactive/Active**

**BTK Y591S Inactive/Active**

**BTK Y598C Inactive/Active**

**BTK Y598D Inactive/Active**

**KIT 1T45_WT Inactive**

**KIT 1T46_WT Inactive**

**KIT 1PKG_WT Active**

**KIT A814S Inactive/Active**

**KIT A814T Inactive/Active**

**KIT A829P Inactive/Active**

**KIT C687S Inactive/Active**

**KIT C691S Inactive/Active**

**KIT D812H Inactive/Active**

**KIT D816E Inactive/Active**

**KIT D816F Inactive/Active**

**KIT D816G Inactive/Active**

**KIT D816H Inactive/Active**

**KIT D816I Inactive/Active**

**KIT D816N Inactive/Active**

**KIT D816V Inactive/Active**

**KIT D816Y Inactive/Active**

**KIT D820E Inactive/Active**

**KIT D820G Inactive/Active**

**KIT D820H Inactive/Active**

**KIT D820N Inactive/Active**

**KIT D820V Inactive/Active**

**KIT D820Y Inactive/Active**

**KIT E583K Inactive/Active**

**KIT E839K Inactive/Active**

**KIT F580C Inactive/Active**

**KIT F584C Inactive/Active**

**KIT F584L Inactive/Active**

**KIT G601R Inactive/Active**

**KIT G664R Inactive/Active**

**KIT G812V Inactive/Active**

**KIT I653T Inactive/Active**

**KIT K550I Inactive/Active**

**KIT K642E Inactive/Active**

**KIT K818R Inactive/Active**

**KIT L656P Inactive/Active**

**KIT N822H Inactive/Active**

**KIT N822K Inactive/Active**

**KIT N822T Inactive/Active**

**KIT N822Y Inactive/Active**

**KIT P627L Inactive/Active**

**KIT R791G Inactive/Active**

**KIT R796G Inactive/Active**

**KIT R804W Inactive/Active**

**KIT S590N Inactive/Active**

**KIT T670E Inactive/Active**

**KIT T670I Inactive/Active**

**KIT T801I Inactive/Active**

**KIT T847P Inactive/Active**

**KIT V559A Inactive/Active**

**KIT V559D Inactive/Active**

**KIT V654A Inactive/Active**

**KIT V825A Inactive/Active**

**KIT V825I Inactive/Active**

**KIT Y823C Inactive/Active**

**KIT Y823D Inactive/Active**

**KIT Y823N Inactive/Active**

**MET 2G15_WT Inactive**

**MET 1R1W_WT Inactive**

**MET 1R0P_WT Inactive**

**MET A1209G Inactive/Active**

**MET D1180N Inactive/Active**

**MET D1228H Inactive/Active**

**MET D1228N Inactive/Active**

**MET D1246H Inactive/Active**

**MET D1246M Inactive/Active**

**MET D1246V Inactive/Active**

**MET D1265Y Inactive/Active**

**MET G1137V Inactive/Active**

**MET H1094L Inactive/Active**

**MET H1094R Inactive/Active**

**MET H1094Y Inactive/Active**

**MET H1106D Inactive/Active**

**MET H1112L Inactive/Active**

**MET H1112R Inactive/Active**

**MET H1112Y Inactive/Active**

**MET H1124D Inactive/Active**

**MET I1053T Inactive/Active**

**MET K1244R Inactive/Active**

**MET K1262R Inactive/Active**

**MET L1097V Inactive/Active**

**MET L1195V Inactive/Active**

**MET L1213V Inactive/Active**

**MET M1131T Inactive/Active**

**MET M1149T Inactive/Active**

**MET M1250I Inactive/Active**

**MET M1250T Inactive/Active**

**MET M1268I Inactive/Active**

**MET M1268T Inactive/Active**

**MET N1118Y Inactive/Active**

**MET Q1067K Inactive/Active**

**MET S1254N Inactive/Active**

**MET T1096S Inactive/Active**

**MET T1173I Inactive/Active**

**MET T1191I Inactive/Active**

**MET T1261A Inactive/Active**

**MET V1092I Inactive/Active**

**MET V1188L Inactive/Active**

**MET V1220I Inactive/Active**

**MET V1290L Inactive/Active**

**MET Y1230C Inactive/Active**

**MET Y1230D Inactive/Active**

**MET Y1230H Inactive/Active**

**MET Y1248C Inactive/Active**

**MET Y1248H Inactive/Active**

**MET Y1253D Inactive/Active**

**RET 1XPD_WT Inactive**

**RET 2IVS_WT Active**

**RET 2IVT_WT Active**

**RET 2IVU_WT Active**

**RET 2IVV_WT Active**

**RET 2X2K_WT Active**

**RET 2X2L_WT Active**

**RET 2X2M_WT Active**

**RET A876V Inactive/Active**

**RET A883F Inactive/Active**

**RET A883P Inactive/Active**

**RET A919V Inactive/Active**

**RET D925H Inactive/Active**

**RET E762Q Inactive/Active**

**RET E768D Inactive/Active**

**RET E884K Inactive/Active**

**RET E901K Inactive/Active**

**RET E921K Inactive/Active**

**RET F893L Inactive/Active**

**RET G748C Inactive/Active**

**RET G894S Inactive/Active**

**RET G911D Inactive/Active**

**RET K907E Inactive/Active**

**RET L790F Inactive/Active**

**RET M918T Inactive/Active**

**RET M980T Inactive/Active**

**RET P766S Inactive/Active**

**RET P973L Inactive/Active**

**RET R749T Inactive/Active**

**RET R813Q Inactive/Active**

**RET R844L Inactive/Active**

**RET R873Q Inactive/Active**

**RET R897Q Inactive/Active**

**RET R908K Inactive/Active**

**RET R972G Inactive/Active**

**RET R982C Inactive/Active**

**RET S765P Inactive/Active**

**RET S767R Inactive/Active**

**RET S891A Inactive/Active**

**RET S922F Inactive/Active**

**RET S922Y Inactive/Active**

**RET T946M Inactive/Active**

**RET V778I Inactive/Active**

**RET V804L Inactive/Active**

**RET V804M Inactive/Active**

**RET Y791F Inactive/Active**

**RET Y826S Inactive/Active**

**Table S3. Analysis of correlated motions in protein kinases**

| **Kinase Region** | **Correlation coefficient** |
| --- | --- |
| **P-loop, all** | **0.58** |
| C-helix, all | **0.82** |
| Hinge loop, all | **0.78** |
| Catalytic loop, all | **0.69** |
| Activation loop, all | **0.98** |
| P-loop, C-helix | **0.38** |
| P-loop, hinge loop | **0.81** |
| P-loop, catalytic loop | **0.83** |
| P-loop, activation loop | **0.95** |
| C-helix, hinge loop | **0.61** |
| C-helix, catalytic loop | **0.48** |
| C-helix, activation loop | **0.94** |
| Hinge loop, catalytic loop | **0.96** |
| Hinge loop, activation loop | **0.75** |
| Catalytic loop, activation loop | **0.69** |
